# Photocatalytic Disinfection of Selected Waterborne Pathogens by Visible Light-Active Nano Iron-Doped TiO_2_ Obtained by a Sol–Gel Method

**DOI:** 10.1021/acsanm.5c01408

**Published:** 2025-04-09

**Authors:** Najeebullah Channa, Tanveer A. Gadhi, Francesca Stefania Freyria, Alessandro Chiadò, Nicola Blangetti, Nicoletta Ditaranto, Barbara Bonelli

**Affiliations:** † Department of Applied Science and Technology, Politecnico di Torino, Corso Duca degli Abruzzi 24, 10129 Torino, Italy; ‡ US Pakistan Center for Advanced Studies in Water (USPCASW), Mehran University of Engineering and Technology, Jamshoro 76062, Pakistan; § INSTM-Unit of Torino Politecnico, Politecnico di Torino, Corso Duca degli Abruzzi 24, 10129 Torino, Italy; ∥ PolitoBIOMed Laboratory, Politecnico di Torino, Corso Duca degli Abruzzi 24, 10129 Torino, Italy; ⊥ Chemistry Department, Aldo Moro University of Bari, Via Orabona 4, Bari 70126, Italy

**Keywords:** photocatalysis, iron-doped TiO_2_ nanoparticles, disinfection, *E. coli*, *S. aureus*, drinking water

## Abstract

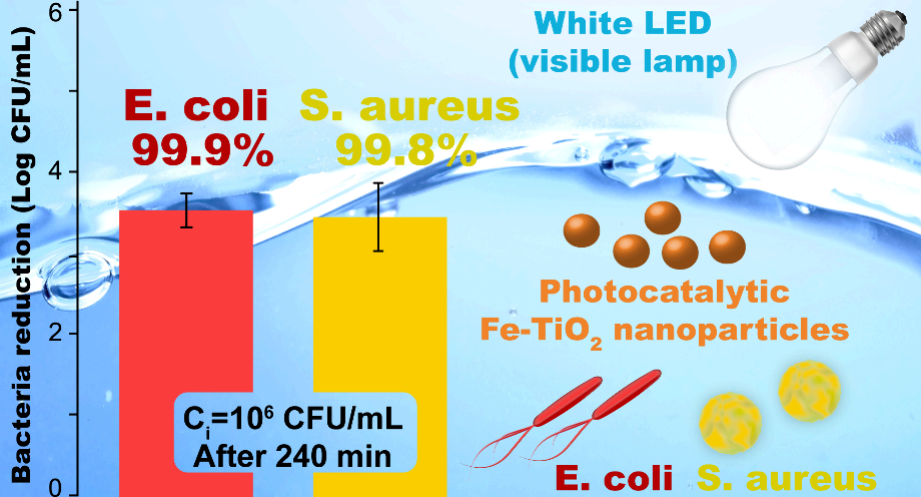

Bacterial contamination in drinking water systems poses a serious health risk due to poor hygiene, human activities, and cross-contamination within the water supply. This study examines the potential of iron-doped titanium oxide nanometric powder (Fe-TiO_2_) for the photocatalytic disinfection of Gram-negative **E. coli** and Gram-positive *S. aureus* under visible light. The Fe-TiO_2_ photocatalyst, with an optimal nominal content of 2.5 wt % Fe, was synthesized using a surfactant-assisted sol–gel method, resulting in a mesoporous nanomaterial composed of anatase nanoparticles with a specific surface area of 123 m^2^/g. A sample of undoped anatase TiO_2,_ obtained using the same sol–gel method and exhibiting a specific surface area of 116 m^2^/g, was utilized to confirm the role of Fe-doping in disinfection. The nanopowders were characterized using X-ray diffraction, N_2_ sorption at −196 °C, diffuse reflectance UV–vis spectroscopy, X-ray photoelectron spectroscopy, electrophoretic mobility measurements, high-resolution transmission electron microscopy combined with energy-dispersive X-ray spectroscopy, and field emission scanning electron microscopy. Photocatalytic disinfection tests were conducted using 1 and 0.5 g/L Fe-TiO_2_ with varying initial bacterial concentrations, with 1 g/L yielding the most promising results under the experimental conditions employed. After 240 min of treatment with 1 g/L Fe-TiO_2_, a 99.9% removal of both **E. coli** and *S. aureus* was achieved starting from a bacterial concentration of 1 × 10^6^ CFU/mL. A 99.9% removal of **E. coli** and a 99.8% removal of *S. aureus* were achieved starting from 1 × 10^4^ CFU/mL. The Fe-TiO_2_ nanomaterial was effective against high concentrations of both bacteria under visible light. Reusability was studied by recovering the Fe-TiO_2_ nanoparticles and assessing their performance over three cycles. The photocatalytic disinfection effectiveness of Fe-TiO_2_ nanoparticles under visible light was validated using an actual tap water sample containing 167 CFU/mL *total coliforms* and 8 CFU/mL **E. coli**. The bacteria were photocatalytically inactivated within 30 min.

## 1 Introduction

The availability of clean water is a significant issue in low- and middle-income countries, where pollutants, particularly pathogenic bacteria, are present in drinking water systems and networks, leading to severe health problems for humans^1,2^Anthropogenic activities and cross-contamination in water bodies such as rivers, lakes, and ponds^3,4^ from sewer lines are considered the primary sources of pathogens in the water supply distribution systems and the resulting waterborne diseases.^5−7^

The presence of various pathogenic bacteria in drinking water, such as *Salmonella* sp., *Shigella* sp., *V. cholera*, **P. aeruginosa**, **E. coli*,* and *S. aureus*, can lead to serious health complications.^8−10^

Conventional water disinfection methods, such as UV light treatment, ozonation, and chlorination, are often expensive, may lead to the formation of toxic byproducts, and require proper technical attention in water treatment plants^11,12^ Specifically, ozonation is quite costly and may produce carcinogenic byproducts in the treated water,^13^ and the disinfection of pathogenic bacteria using high doses of chlorine can negatively impact human health and the environment.^14,15^ Furthermore, some residual microbial contamination can still be found even after applying these treatments.^16^ Therefore, alternative and effective water disinfection methods are necessary to eliminate pathogenic bacteria. In this context, heterogeneous photocatalysis, a type of advanced oxidation process (AOP),^16^ could enable the simultaneous inactivation of pathogenic bacterial cells and the degradation of other organic pollutants.

The use of photocatalysis for water disinfection is emerging as a promising alternative to conventional disinfection methods, particularly when sunlight can be exploited.^17^ With increasing interest in photocatalytic disinfection, several authors are exploring the potential of new nanomaterials with upconversion and plasmonic properties, which can efficiently harness longer wavelength light in the visible and NIR ranges.^18,19^

TiO_2_ is one of the widely used photocatalysts for water remediation,^20^ alongside other semiconductors like ZnO. TiO_2_-based photocatalysts can inactivate several Gram-negative and Gram-positive bacteria,^21^ as well as degrade and even mineralize numerous organic contaminants at ambient temperature and pressure.^22,23^

The photocatalytic disinfection of **E. coli** and *S. aureus* using TiO_2_ has been reported under UV light.^24^ Indeed, one of the significant disadvantages of TiO_2_ is its large band gap, necessitating the use of UV light, which constitutes a minor fraction of the solar spectrum. To address this limitation, doping with metals such as Fe, Cu, Zn, or Cd,^25−28^ can decrease TiO_2_ band gap, shifting its absorption edge toward the visible range. The lifetime of photogenerated electrons and holes is another drawback of TiO_2_: metal doping (for instance, with Fe) can also enhance charge separation between holes (h^+^) formed in the valence band and electrons (e^–^) promoted to the conduction band.^29^

Among suitable heteroatoms for doping TiO_2_, iron is the most abundant element on Earth overall and the fourth most abundant element in the crust^30^; it also plays a crucial role in several biological and chemical processes.^12^ Fe-doped mesoporous TiO_2_ nanoparticles, obtained in our laboratory through a direct doping method using surfactant-assisted sol–gel techniques, have demonstrated promising photocatalytic activity under simulated solar light for degrading phenol, a recalcitrant contaminant, and Acid Orange 7, a model molecule for nitrogen-containing organic pollutants.^31−33^ The optimal Fe content was determined to be 2.5 wt %, because at higher Fe content, surface defects formed, resulting in undesired recombination of photogenerated charge carriers.^34^

Based on these results, this study reports the disinfection efficacy of Fe-doped TiO_2_ nanoparticles (with a nominal iron content of 2.5 wt %) under visible light against two types of bacteria strains: Gram-negative **E. coli** and Gram-positive *S. aureus*.**E. coli** is commonly found in contaminated drinking water and food and is a leading cause of diarrhea and hemolytic uremic syndrome; *S. aureus* is a primary pathogen associated with hospital-acquired infections and quickly develops antibiotic resistance.^35−37^

Table S1 reports relevant literature on photocatalytic bacteria disinfection using Fe-doped TiO_2_-based materials.^29,34,45−48,35,38−44^ Several of these papers imply the fabrication of composites, the codoping of Fe-TiO_2_ with other elements, or employing UV light for disinfection.^29,49^

Regarding the disinfection properties of Fe-doped TiO_2_ under visible light, we found only two papers focused on powders^34,38^ and two others on thin films.^35,46^ Among these, only one paper suggests the use of Fe-doped TiO_2_ against both **E. coli** and *S. aureus*: Yadav et al.^38^ studied the photocatalytic disinfection of **E. coli** and *S. aureus* using 1–3 mol % Fe-TiO_2_ (i.e., an Fe content comparable to the sample studied here) under the irradiation of eight fluorescent lamps (Philips TLD 8 W, λ mainly >400 nm with low emission in the near UV range). Khan et al.^34^ investigated the inactivation of **E. coli** using 0.1 wt % Fe-TiO_2_ under visible light irradiation using a halogen lamp 500 W at a light intensity of 30,798 lx.

We want to emphasize that the preparation method may significantly affect the surface and structural properties of Fe-doped TiO_2_. The Fe content is only one factor to consider when evaluating photocatalytic efficiency, as other parameters, such as the type of TiO_2_ polymorph, nanoparticle size, porosity, and surface composition, may also play a role.^31,32^ From the photocatalytic perspective, anatase is regarded as one of the most active TiO_2_ polymorphs, due to its high surface area and indirect band gap, which inhibits electron–hole recombination.^50^ In this study, the photocatalysts were synthesized using a template-assisted sol–gel method, resulting in 100% anatase phase TiO_2_ nanoparticles with a uniform size (approximately 10 nm, vide infra), high specific surface area, and effective Fe inclusion.^31−33^ Another essential aspect of disinfection is that the rate can vary depending on the bacterial strains, likely because different bacteria possess different protection mechanisms.^51^ Moreover, strains isolated from freshwater and wastewater exhibit greater resistance to AOPs than pure-type strains.^52^ Pure strain tests are typically performed in defined solutions, such as saline solutions, known buffers, and culture media. However, actual water samples have different characteristics and contain various anions (Cl^–^, F^–^, NO_3_^–^, SO_4_^2–^, etc.) and metal cations (Ca^2+^, Mg^2+^, K^+^, Zn^2+^, Fe^3+^, etc.) that could affect the performance of the chosen disinfection process.^53^

In addition, there is currently no recommendation regarding the concentration of the targeted microorganisms that would yield relevant outcomes easily applicable to real-life scenarios. Additionally, the bacterial load can vary from a few to 10^6^ CFU/mL depending on the sampling point.^54^ This variability is critical, as the amount of photocatalytic nanomaterial must be adjusted according to the starting concentration of the bacteria.^55^ Typically, the initial bacterial concentration is determined based on the contamination level of tap water. *Total coliform, fecal coliform*, and **E. coli** are regarded as key indicators of contamination in aquatic environments.^56,57^

The tests were performed with two different starting concentrations of bacteria, 10^6^ and 10^4^ CFU/mL, to replicate two levels of contamination. In both scenarios, the rate of bacterial inactivation was monitored for at least 4 h using the colony count method, which is a commonly employed technique for determining the number of viable cells.^58^

Finally, to demonstrate the real applicability and potential of the nanomaterials, a sample of contaminated tap water was collected from a household in the Jamshoro region of Pakistan: the sample was microbiologically characterized and tested for its photocatalytic disinfection.

## 2 Experimental Section

### 2.1 Reagents

ACS-grade chemicals were used: most of the chemicals for the TiO_2_ synthesis were acquired from Merck-Sigma-Aldrich (Schnelldorf, Germany); ethanol was acquired from Merck, Sigma-Aldrich (Italy), and sodium chloride (NaCl, 99.5%) from Daejung (Daejung chemicals and metals, China).

For bacterial analysis, Luria–Bertani broth (LB), Muller Hinton agar (MH), and RAPID’ **E. coli** agar were acquired from Oxoid (England). To investigate cell damage, the LIVE/DEAD BacLight Bacterial Viability Kit was acquired from Thermo Fisher Scientific (USA).

### 2.2 Synthesis of Fe-Doped and Undoped TiO_2_

**Scheme 1:**
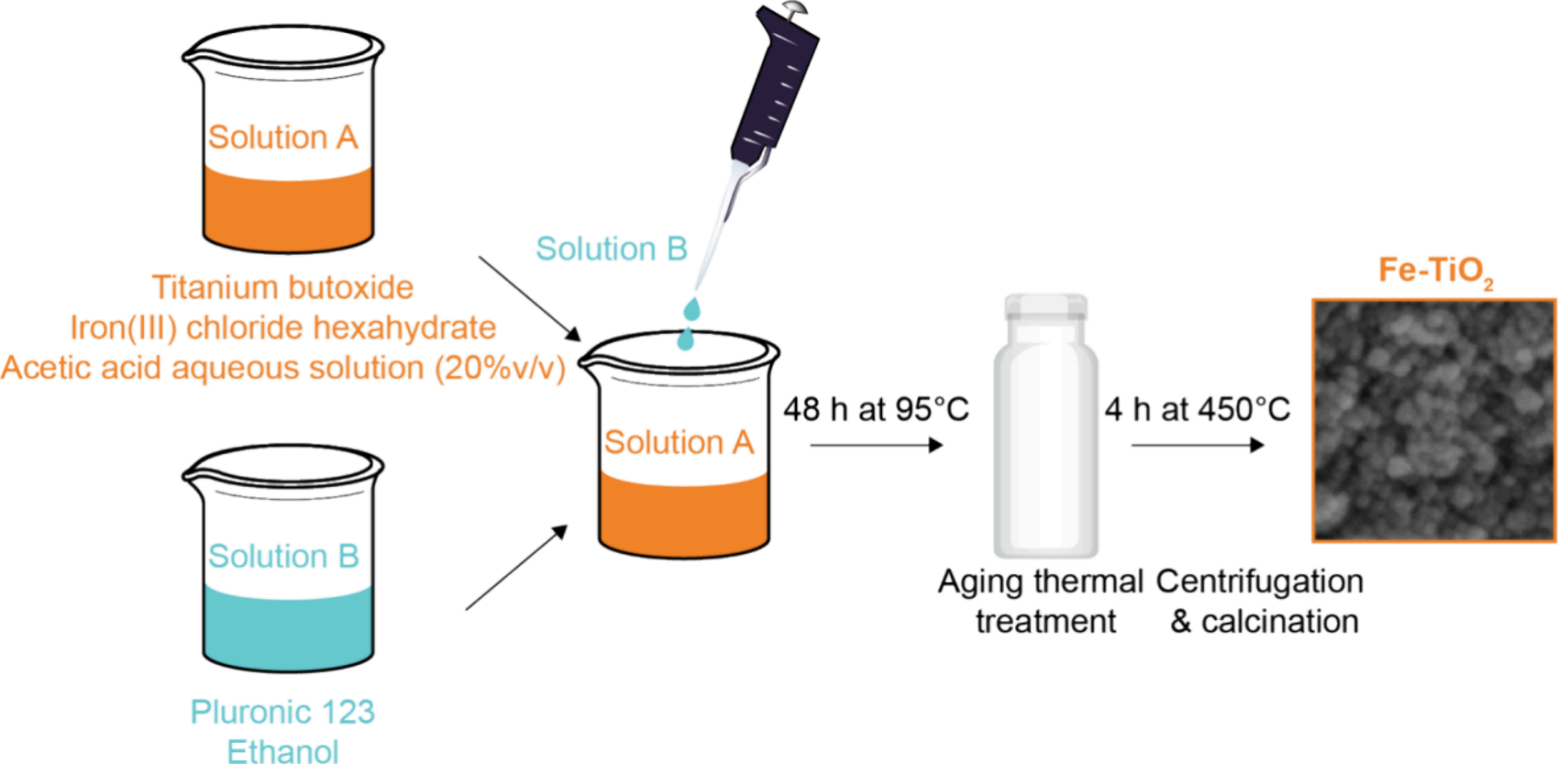
A Scheme of the Synthesis Procedure That We Adopted To Produce the Fe-TiO_2_ Nanoparticles^45,54^

A sample of TiO_2_ with a nominal Fe content of 2.5 wt % (Fe-TiO_2_) was synthesized using the method detailed in ref (32,33) and depicted in Scheme 1.

Two solutions (A and B) were initially prepared. Solution A was obtained by adding approximately 20. g of Ti(OBut)_4_ (titanium butoxide) dropwise to 120 mL of a 20% v/v acetic acid solution with the addition of a nominal amount of FeCl_3_·6H_2_O (iron(III) chloride hexahydrate) corresponding to 2.5 wt % Fe. The solution was stirred for 4 h. Meanwhile, solution B was prepared by adding approximately 12 g Pluronic P123 (poly(ethylene glycol)-*block*-poly(propylene glycol)-*block*-poly(ethylene glycol)) to 80 mL of ethanol. Solution B was added dropwise to solution A and stirred continuously for 24 h at room temperature to produce a neat and transparent solution. Finally, the obtained mixture was transferred into a Teflon autoclave for hydrothermal treatment at 95 °C for 48 h. The resulting precipitate was centrifuged at 8,000 rpm for 15 min and washed twice with ethanol and water. Afterward, it was dried at 80 °C in a static stove and then calcined in air at 450 °C for 4 h (heating and cooling rate: 1.8 °C/min). A sample of undoped TiO_2_ was synthesized using the same procedure, omitting the addition of FeCl_3_·6H_2_O.

### 2.3 Physicochemical Characterization of the Powders

The phase composition and crystallinity of the powders were analyzed by collecting their X-ray diffraction (XRD) patterns on an X’Pert Philips PW3040 (Panalytical, Almelo, Netherlands). The X’Pert High Score Plus 3.0e software (Malvern Panalytical Ltd., Malvern, UK) was used to analyze the patterns.

N_2_ adsorption/desorption isotherms were measured at −196 °C on nanopowders that had been previously outgassed at 150 °C to eliminate water and other atmospheric contaminants (Quantachrome Autosorb 1C, Boyton Beach, FL, USA). The Specific Surface Area (SSA) of the samples was obtained using the BET (Brunauer–Emmett–Teller) method. The Barrett–Joyner–Halenda (BJH) method was applied to the desorption branch of the isotherm for *P*/*P*^0^ values exceeding 0.35 to determine the pore size distribution of the samples.

The diffuse reflectance (DR) UV–vis spectra of the samples were measured using a Varian Cary 5000 UV–Vis–NIR spectrophotometer (Agilent, Milan, IT) equipped with a DR sphere to analyze powders.

The surface chemical composition and speciation were analyzed using X-ray photoelectron spectroscopy (XPS) with a Versa Probe II scanning XPS microprobe spectrometer (Physical Electronics GmbH) equipped with a monochromatized Al K_α_ source (X-ray spot = 200 μm) at a power of 50.9 W. Both wide scans and high-resolution XP spectra were obtained using a fixed analyzer transmission (FAT) mode with pass energies of 117.40 and 29.35 eV, respectively. An electron gun facilitated charge compensation (1.0 V, 20.0 μA). All binding energy (BE) values were referenced to the C 1s line at 284.8 ± 0.1 eV for adventitious carbon. Data processing was completed using MultiPak software version 9.9.0.8.

The zeta potential of the nanopowders was measured using a Zetasizer Nano-ZS (Malvern Instruments, Worcestershire, UK). The nanoparticles were suspended in Milli-Q water and stirred magnetically for 5 min. Subsequently, the pH was adjusted by adding 0.1 M HCl or 0.1 M NaOH solutions.

The morphology of the nanopowders was investigated using electron microscopy techniques. Transmission Electron Microscopy (TEM) analysis was performed using a TALOS F200X (Thermo Scientific) microscope operated at 200 kV. TEM images were captured with a 16 Mpx CMOS camera (Ceta, Thermo Scientific). Scanning TEM (STEM) images were obtained on the same machine using an electron beam set at 200 kV and a current of approximately 25 pA in HAADF (High-Angle Annular Dark Field) mode.

Compositional maps were obtained using Energy Dispersive X-ray Spectroscopy (EDS). To achieve this, the probe current of the 200 kV electron beam was raised to approximately 750 pA. Simultaneously, the signal was collected by a 4-quadrant Silicon Drift Detector (SDD) and processed using the machine acquisition software (Velox, v. 3.10, Thermo Scientific) to extract elemental mapping. At least two maps for each sample were collected, and the corresponding EDS spectra were extracted from the entire map.

A Field Emission Scanning Microscope (FE-SEM) from MERLIN ZEISS AG, Oberkochen, Germany, was used to investigate further the morphology of the nanopowders (Figure S1).

The iron content in the Fe-TiO_2_ nanopowder was measured using ICP/MS (Inductively Coupled Plasma Mass Spectrometry) on an ICAP Q (Thermofisher) before and after exposure to the bacteria cultures (vide infra). Approximately 10 mg of the sample was weighed and dissolved in a 25 mL solution. To aid dissolution, 2 mL of H_2_SO_4_ was added, and the mixture was heated in a beaker covered with a watch glass until white fumes appeared, ensuring complete dissolution. A four-point calibration curve was obtained using 100, 500, 1000, and 2000 ppb standards for quantification. The sample was subsequently diluted 10 or 50-fold before analysis.

### 2.4 Photocatalytic Disinfection Tests

#### 2.4.1 Preparation of the Bacterial Cultures

**E. coli** (ATCC 8739)^59^ and *S. aureus* (ATCC 25923)^60^ were used. Initially, both bacteria were grown overnight in LB broth at 37 °C for 24 h.^61^ Afterward, each bacterial mixture was centrifuged at 5,000 rpm for 15 min at 25 °C and washed three times with normal saline solution (0.85% w/v). Finally, the bacterial concentration was assessed by optical density (OD) measurement at 600 nm. Then, the bacterial suspension was diluted to adjust the bacterial concentration to either 10^4^ or 10^6^ CFU/mL.^61^ The plate count method, employed in most papers referenced in Table S1, validated the bacterial concentration.

#### 2.4.2 Photocatalytic Disinfection of *E. coli* and *S. aureus* in Liquid Media

The photocatalytic disinfection experiments were performed with both strains at a relatively high bacterial load of 10^6^ or 10^4^ CFU/mL. Typically, 50 mg of Fe-TiO_2_ was added to a beaker containing 50 mL of bacterial mixture (powder concentration of 1 g/L), and the suspension was stirred in the dark for 45 min. During the photocatalytic disinfection tests, the suspension was irradiated with a commercially available white LED lamp (Philips, 1535 lm, emission spectrum ranging from 430 to 800 nm) positioned approximately 45 cm from the bacteria solution^61^ at room temperature (about 25 °C). In each experiment, after 0, 15, 30, 60, 90, 120, and 240 min, a 100 μL volume was withdrawn and serially diluted to 0.85% normal saline solution. Then, 100 μL of the diluted samples were spread on MH agar plates and incubated at 37 °C for twenty-4 h. After incubation, bacterial colonies were counted using a manual colony counter (Isolab, Germany) and reported in CFU/mL.

Three control experiments were included: (i) a “light control”, involving the visible light irradiation of a bacterial suspension without any photocatalyst; (ii) a “dark control 1”, involving the bacterial suspension and Fe-TiO_2_ nanoparticles in the dark, and (iii) a “dark control 2” involving the bacterial suspension without photocatalyst in the dark. For brevity, the results of the “dark control 2” experiments will be reported in the SI.

To validate the antibacterial activity of Fe-TiO_2_ under visible light, the experiments on the 10^6^ CFU/mL mixtures (50 mL) were also conducted in the presence of 50 mg of undoped TiO_2_ (nanopowder concentration of 1 g/L).

#### 2.4.3 Reusability Tests

After the initial experiments, the reusability of Fe-TiO_2_ was investigated. Three cycles were carried out with both bacterial strains to assess the stability and possible reuse of the nanomaterial. Initially, 80 mL of the bacterial and nanopowder mixture (with a powder concentration of 1 g/L) was stirred in a beaker for 45 min in the dark. Subsequently, the solution was irradiated under visible light.

After each cycle, the Fe-TiO_2_ nanoparticles were collected and washed three times in total, twice with 70% ethanol solution and once with Milli-Q water, then centrifuged at 5000 rpm for 15 min at 25 °C to remove residual biomass and any viable remaining bacterial cells. Afterward, the nanoparticles were dried in an oven at 80 °C^62^ for 5 h and then used for another inactivation test under the same conditions.

In each experiment, after 0, 120, and 240 min, a volume of 100 μL was withdrawn and serially diluted to 0.85% normal saline solution. Then, 100 μL of the diluted samples was spread on MH agar plates and incubated at 37 °C for 24 h. After incubation, bacterial colonies were counted and reported in CFU/mL.

#### 2.4.4 Photocatalytic Disinfection Tests of Actual Tap Water Samples

The disinfection potential of Fe-TiO_2_ nanoparticles under visible light was also tested with actual tap water collected from a household near Mehran University in Jamshoro, Pakistan. A tap water sample (approximately 1 L) was collected in a sterilized sample bottle and transported to the laboratory for further physicochemical and microbial analyses.

Initially, the total dissolved solids (TDS) and pH of the tap water were measured using portable instruments (Hanna EC/TDS meter Hi99301 and Hanna pH meter H18424). Sulfate and nitrate concentrations were analyzed using a UV–vis spectrophotometer (Shimadzu, 1800) following the 4500-SO_4_^2–^ and 4500-NO_3_^–^ standard methods recommended by the American Public Health Association (APHA). Chloride concentration and total hardness were measured in accordance with the guidelines of APHA standard methods.^63^

Specific agar (RAPID’ **E. coli** agar) was used for the microbial analysis of **E. coli** and *total coliform*.^8,64^ Initially, 100 mL of the tap water sample was passed through a membrane filter (mixed cellulose ester membrane, 0.45 μm); then, the filter was placed on an agar plate and incubated at 37 °C for 24 h. Afterward, the bacterial colonies were counted using a colony counter and reported as CFU/mL units.

Since the tap water sample showed some bacterial contamination (Table S2), 100 mL of tap water was mixed with 100 mg of Fe-TiO_2_ to achieve the same concentration of 1 g/L of the experiments carried out with the bacterial strains in liquid media: the suspension was irradiated under visible light as reported in 2.4.2. After 15 min of stirring in the dark, photocatalytic disinfection was carried out under the LED lamp at room temperature.

To assess the bacterial disinfection, 1 mL of suspension (tap water and photocatalyst) was withdrawn at different time intervals of 0, 15, and 30 min, spread on specific RAPID’ **E. coli** agar,^8^ and incubated at 37 °C for 24 h. Finally, the bacterial colonies were counted using a manual colony counter and reported in CFU/mL.

#### 2.4.5 Bacterial Cells Live/Dead Staining and Fluorescence Microscopy

Live/dead staining was carried out and analyzed by fluorescence microscopy to assess cell viability. Initially, 1 mL aliquots were withdrawn from the treated suspension at different intervals, followed by centrifugation at 10,000 × *g* for 10 min to obtain the bacterial pellets. The pellets were washed three times with a 0.85% normal saline solution, after which the supernatant was drained. Subsequently, the pellets were resuspended in 1 mL of 0.85% of NaCl and mixed. Then 5 μL of mixed dyes (SYTO 9 and propidium iodide (PI), 1:1(v:v)) was added to stain the bacterial suspension and incubated in the dark for 15 min at room temperature. Afterward, a 5 μL sample was pipetted onto a glass slide and analyzed using an Axio Scope A1 fluorescence microscope (Zeiss, Germany).^61^

## 3 Results and Discussion

### 3.1 Physicochemical Characterization of the Undoped and Fe-Doped TiO_2_ Powders

**Figure 1:**
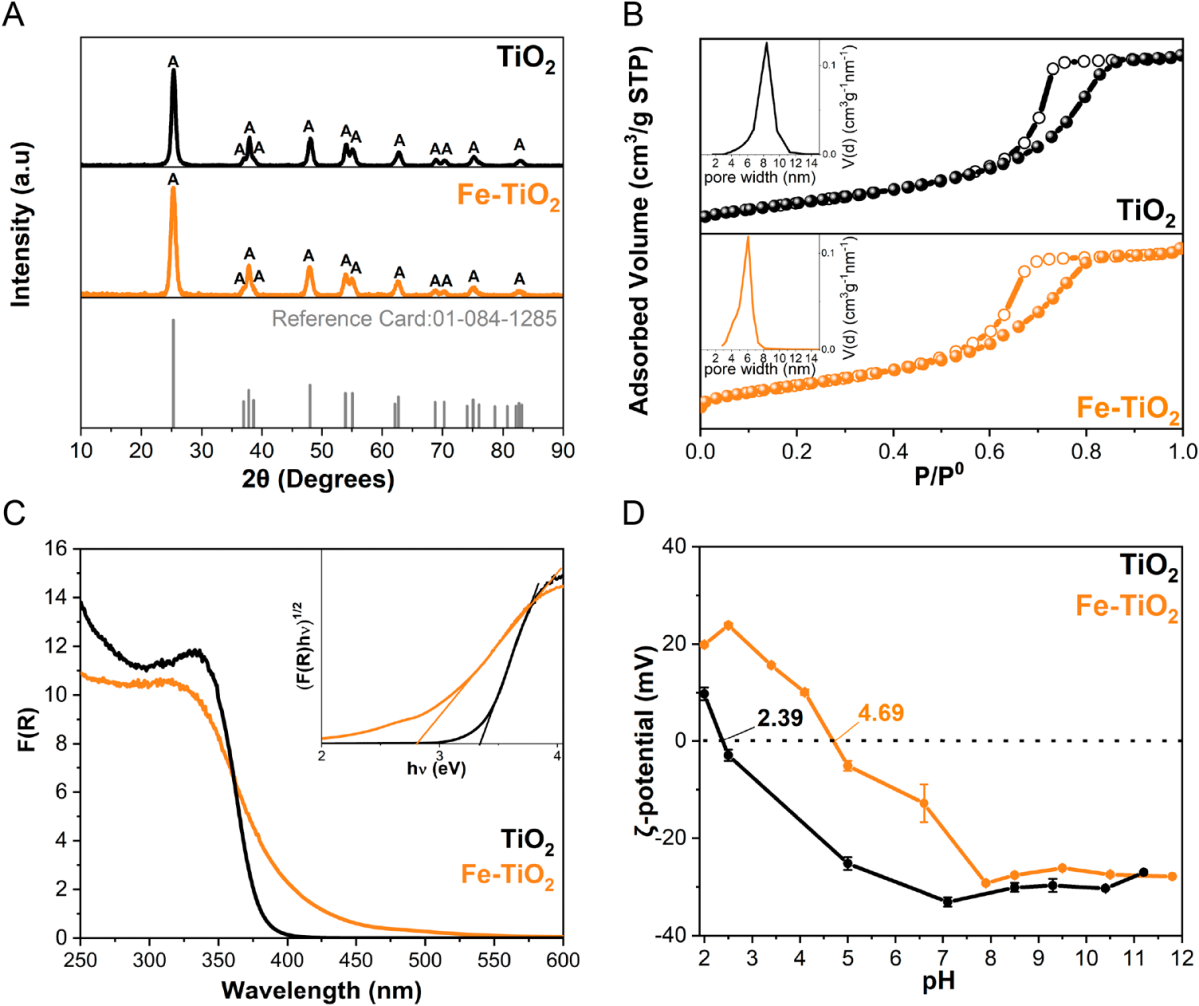
(A) XRD patterns of undoped TiO_2_ (black line) and Fe-TiO_2_ (orange line) nanopowders; the vertical bars correspond to the anatase peaks in the 01-084-1285 reference card. (B) N_2_ adsorption/desorption isotherms at −196 °C of undoped TiO_2_ (black line) and Fe-TiO_2_ (orange line) nanopowders; full and empty symbols represent adsorption and desorption branches, respectively. The insets show the corresponding PSD, as determined by the BJH method; (C) DR UV–vis spectra of undoped TiO_2_ (black line) and Fe-TiO_2_ (orange line) nanopowders; the inset shows the corresponding Tauc’s plot. (D) ζ-Potential curves based on electrophoretic mobility measurements versus pH of undoped TiO_2_ (black line) and Fe-TiO_2_ (orange line) nanopowders.

The crystalline phases of both Fe-TiO_2_ and undoped TiO_2_ were characterized using powders XRD. As shown in Figure 1A, for both powders, all observed peaks correspond to the TiO_2_ anatase phase (reference PDF card: 01-084-1285). Consistent with our previous work on Fe-doped TiO_2_ nanoparticles with similar Fe content,^31,32^ XRD did not detect peaks ascribable to any crystalline Fe-containing phases, likely due to the low Fe content.

The average crystallite size, determined by applying Debye–Scherrer’s method, was 11 ± 3 and 14 ± 3 nm for Fe-TiO_2_ and undoped TiO_2_, respectively. The slightly smaller crystallite size of the Fe-doped sample may be due to the presence of Fe, which inhibits crystallite growth, as previously reported in the literature.^32^

The N_2_ sorption isotherms at −196 °C are shown in Figure 1B. Both powders exhibit type IV isotherms, characteristic of mesoporous materials with very limited microporosity^65^ Both isotherms show an H2(b) hysteresis loop, which may result from some delayed condensation and desorption pore-blocking effects within inkbottle mesopores. The specific surface area (SSA) and total pore volume (*V*_tot_) values are reported in Table 1: a slight increase in both values was observed with Fe-doping, alongside a decrease in the most probable pore size (inset to Figure 1B), as determined by the BJH method.

**Table 1:** Relevant Physicochemical Properties of the Samples

sample	average crystallite size (nm)^a^	SSA (m^2^ g^–1^)^b^	*V*_tot_ (cm^3^ g^–1^)^b^	pH_IEP_^c^	bandgap energy (*E*_g_, eV)^d^	valence band energy (VB, eV)^e^	conduction band energy (eV)^f^
undoped TiO_2_	14 ± 3	116	0.18	2.39	3.34	2.60	–0.74
Fe-TiO_2_	11 ± 3	123	0.21	4.69	2.80	2.30	–0.50

a As determined by applying the Debye-Scherr method to the XRD patterns.

b As determined by N_2_ sorption isotherms at −196 °C.

c As determined by electrophoretic measurements.

d As determined by applying Tauc’s plot method for indirect semiconductors.

e As determined by XPS.

f Calculated as CB = VB – *E*_g_.

The DR UV–vis spectra obtained with the two nanopowders are shown in Figure 1C. With undoped TiO_2_, the typical absorption band due to the O^2–^ to Ti^4+^ charge transfer transition is observed below 400 nm. In comparison to undoped TiO_2_, the Fe-TiO_2_ UV–vis spectrum shows (i) a red-shift of the absorption onset, due to Fe doping, and (ii) a broad absorption band at longer wavelengths (approximately 500 nm), ascribed to the *d–d* transition of Fe^3+^ ions in some surface Fe-oxyhydroxide species (FeO_*x*_H_*y*_), as already discussed in references.^31,32^ The amount of such FeO_*x*_H_*y*_ species is limited, and/or they are likely highly dispersed at the surface, ultimately eluding XRD detection.

The samples’ bandgap was evaluated using Tauc’s plot method for indirect semiconductors (i.e., by plotting (*F*(*R*)*hυ)^1/2^ versus Energy, eV) being anatase the only polymorph. The inset in Figure 1C shows the corresponding Tauc’s plots from which band gap energy (*E*_g_) values of 3.34 eV (λ_max_ = 371 nm) and 2.80 eV (λ_max_ = 443 nm) were extrapolated for undoped TiO_2_ and Fe-TiO_2_, respectively. These results suggest an improved light-capturing ability due to Fe-doping, which may lead to improved photocatalytic activity of Fe-TiO_2_ in the visible range, attributed to both the red-shift of the bandgap and the presence of surface FeO_*x*_H_*y*_ species that absorb in the visible range.

XPS enabled the measurement of the valence band (VB) energy position (spectra not shown). The corresponding values (Table 1) were employed to determine the CB position by considering the *E*_g_ values determined using DR UV–vis spectroscopy (CB = VB – *E*_g_). In comparison to undoped TiO_2_ (VB = 2.60 eV), the VB shifts to 2.30 eV, confirming Fe doping.^66^

**Scheme 2:**
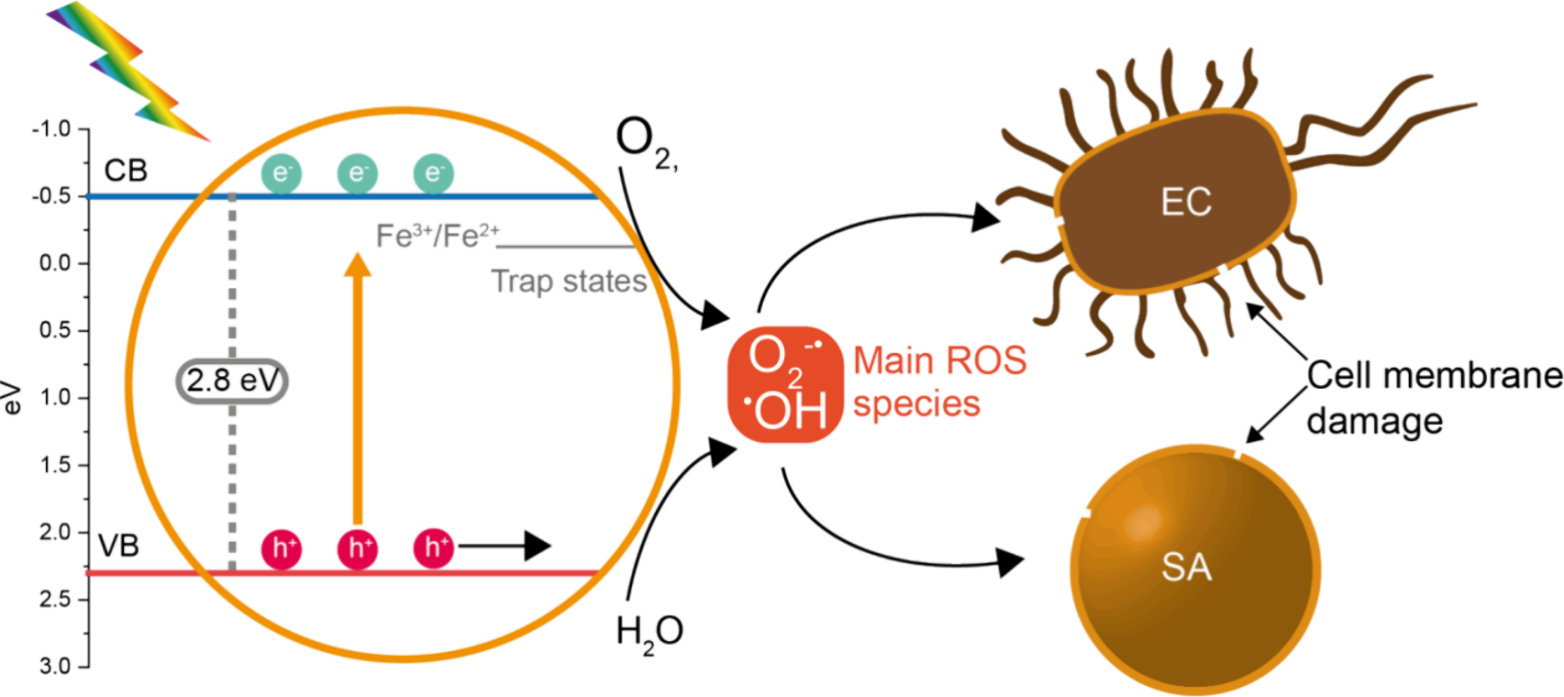
Possible Events Occurring under Visible-Light Illumination of the Studied Fe-TiO_2_ Nanoparticles, Including the Formation of ROS and Subsequent Inactivation of **E. coli** and *S. aureus* Bacterial Cells

The line shape of high-resolution Fe 2p XP spectrum of Fe-TiO_2_ (Figure S2) was compared with the line shape of Fe^3+^ and Fe^2+^ containing compounds and underwent curve-fitting. The thick envelope and the absence of a shakeup feature between 2p3/2 and 2p1/2 peaks, along with the fitting results, suggested the copresence of both Fe^3+^ and Fe^2+^ ions. Due to the complex multiplet arising from the two oxidation states, Figure S2 reports a simplified curve fitting that emphasizes the presence of the peak component at BE = 709.4 ± 0.1 eV (main peak for Fe^2+^) and that at BE = 710.9 ± 0.1 eV (main peak for Fe^3+^). The component at 716 eV can be interpreted as both Fe^3+^ surface peak and Fe^2+^ shakeup.^67^ The sampling depth of the XPS analysis (approximately 7–8 nm) allows for the investigation of surface and subsurface atomic layers. In this case, we can hypothesize that Fe-doping leads to the formation of certain defects, namely Fe^2+^ species, likely located beneath the very first atomic layers, which can also serve as trap sites (as depicted in Scheme 2). These same species could also contribute to the tail observed at longer wavelengths in the DR UV–vis spectrum of Fe-TiO_2_.

Figure 1D presents the electrophoretic mobility measurements of the two powders, both exhibiting a negatively charged surface across a broad pH range. Interestingly, Fe-TiO_2_ shows an increase of the pH at the isoelectric point (pH_iep_), likely due to the presence of surface FeO_*x*_H_*y*_ species detected by DR UV–vis spectroscopy (Figure 1C), which aligns with previous work.^32^

**Figure 2:**
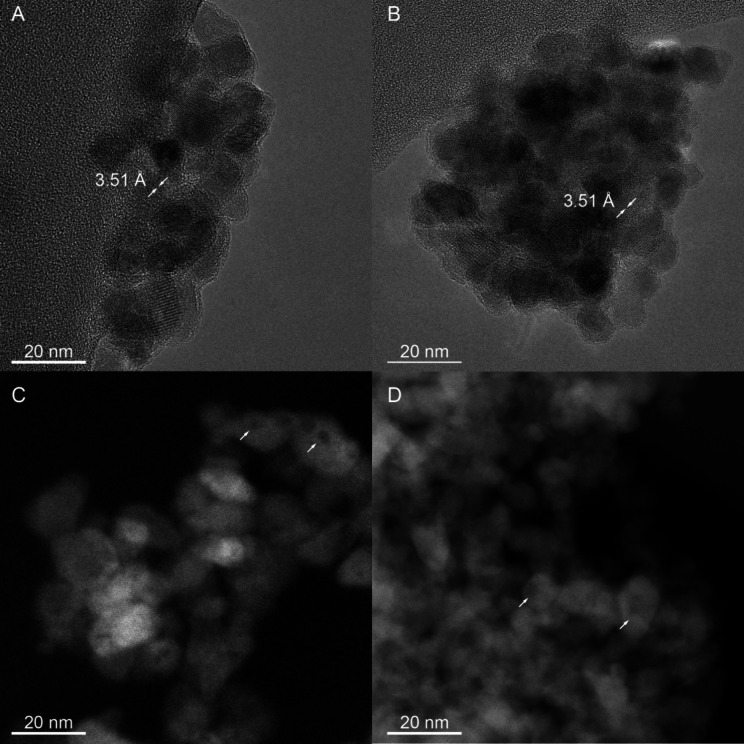
Selected HRTEM images in transmission (A, B) and STEM mode (C, D) of undoped TiO_2_ (panels A and C) and Fe-TiO_2_ (panels B and D).

Figure 2A,B display two selected HRTEM micrographs of the studied nanopowders; both reveal the presence of roundish nanoparticles with a relatively uniform shape and size (in the 10–13 nm range), exhibiting some degree of agglomeration/aggregation. The HRTEM micrographs indicate a lattice spacing of 3.51 Å for both nanopowders, characteristic of the (101) plane of the anatase phase. Notably, the observed nanoparticles’ size is comparable to the crystallite size determined by XRD, suggesting that the employed synthesis method produces monocrystalline nanoparticles. Furthermore, images captured in STEM mode (Figure 2C,D) demonstrate the presence of intraparticle porosity.

Elemental analysis of the Fe-TiO_2_ nanopowder by EDS (Figure S3) confirmed the presence of iron, with an average content of 2.56 wt % ± 0.41%. This aligns with the nominal amount (2.5 wt % Fe). The Fe content, measured by ICP-MS, confirmed the presence of iron at 2.44 wt % (Table S3). The slight discrepancy between the two values may relate to EDS being a semiquantitative technique, while the ICP-MS is a more reliable method for element quantification.

### 3.2 Photocatalytic Disinfection of *E. coli* and *S. aureus* in Liquid Media

Once characterized, Fe-TiO_2_ was tested for the photocatalytic disinfection of selected bacterial strains (*E. coli* and *S. aureus*) at two different bacterial loads (10^6^ and 10^4^ CFU/mL) to simulate two relatively high levels of pathogens found in actual tap water samples. Indeed, although no bacteria should be detected in drinking water (the WHO-recommended threshold is 0 CFU/mL), there are several examples of contamination with bacterial loads ranging from a few to 10^6^ CFU/mL.^54^

**Figure 3:**
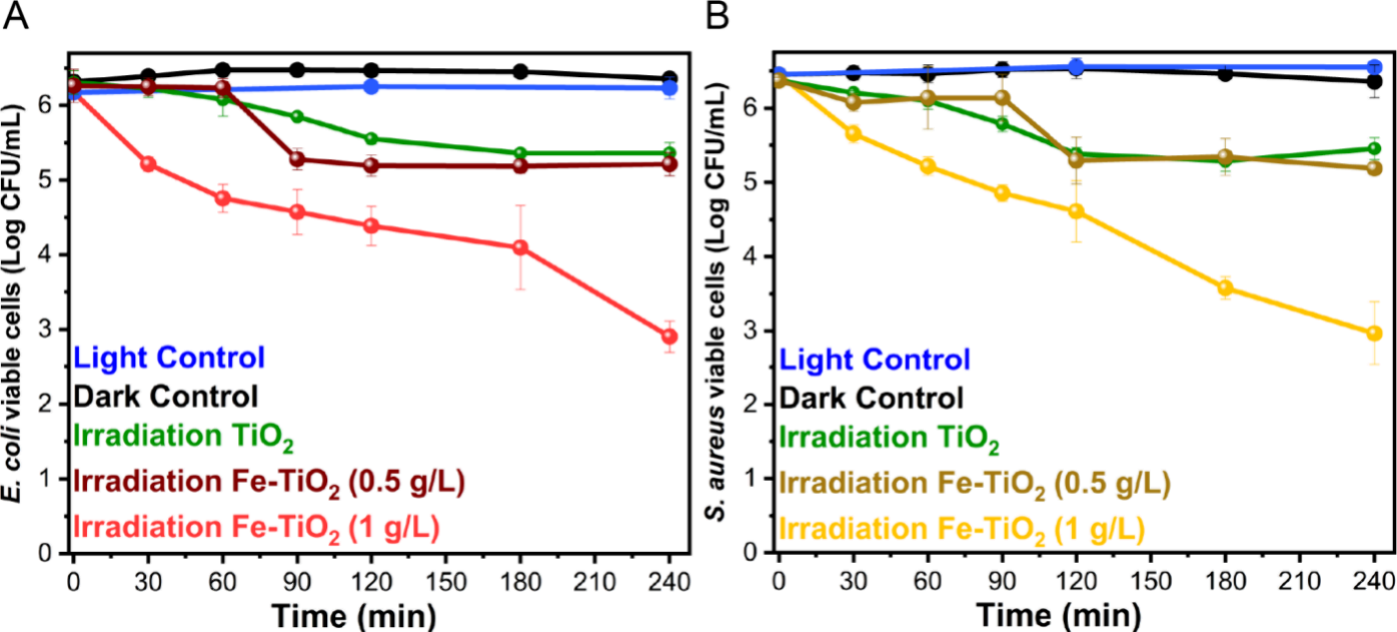
Evaluation of the viable CFU/mL over time (Log scale) for (A) **E. coli** in the presence of 1 g/L Fe-TiO_2_ (red line), 0.5 g/L Fe-TiO_2_ (dark red line), or 1 g/L undoped TiO_2_ (green line) under visible light irradiation, and (B) *S. aureus* in the presence of 1 g/L Fe-TiO_2_ (yellow line), 0.5 g/L Fe-TiO_2_ (light brown line) and 1 g/L undoped TiO_2_ (green line) under visible light irradiation. An initial bacterial concentration of 10^6^ CFU/mL was employed. All the panels also depict the viable CFU/mL over time during the light control experiments (blue line, bacteria suspension without photocatalyst under visible light irradiation) and of the dark control 1 experiments (black lines, bacteria suspension in the presence of 1 g/L Fe-TiO_2_ in the dark).

Figure 3A,B display the results of the disinfection experiments with an initial bacteria concentration of 10^6^ CFU/mL for both *E. coli* and *S. aureus*. In the presence of the visible-light-activated nanomaterials, both strains demonstrated a dramatic decrease in viable bacterial cells (Figures S4 show images of some of the plates used for the counts). Similar results were previously achieved using Bi_2_O_3_ nanomaterial with the same bacterial strains using the same inoculum concentration, i.e., 10^6^ CFU/mL.^61^

The Log reduction in relation to the initial bacterial concentration was calculated to assess the efficiency of bacterial inactivation. This value is generally used to compare the effects of various water treatment processes.^25^ Notably, Fe-TiO_2_ nanoparticles at a concentration of 1 g/L allowed for the reduction of viable bacteria to almost 10^2^ CFU/mL after 240 min of visible light exposure, which corresponds to nearly a 99.97% reduction or 3.53 Log (3.57 ± 0.21 and 3.49 ± 0.42 Log reductions for **E. coli** and *S. aureus*, respectively), whereas TiO_2_ at the same concentration of 1 g/L concentration achieved less than 90% reduction (0.95 ± 0.14 and 0.92 ± 0.15 Log reductions for **E. coli** and *S. aureus*, respectively). Therefore, the Fe-doped nanomaterial was 2.6 times more effective than the undoped TiO_2_ nanopowder, likely due to its absorption properties in the visible range, as identified by DR UV–vis spectroscopy. Conversely, the photocatalytic efficacy of undoped TiO_2_ is limited under visible light, consistent with its optical properties.

The results from the control experiments support the hypothesis that these outcomes are primarily due to the photocatalytic behavior of the studied nanomaterials. The viable bacterial cells of both strains remained nearly stable in the light and dark controls throughout the entire test (the results of dark control 2 are reported in Figure S5) as nearly no growth or significant Log reduction was observed. Indeed, as illustrated in Figure 3, the light control (blue line) indicated no apparent effect (no growth promotion or inhibition) of visible light on the growth of both bacteria compared to dark control 2. Similarly, since dark control 1 allows for the evaluation of any potential toxic effects of the nanomaterial without light activation, the stable values of CFU/mL obtained during these control experiments confirm that the nanomaterial toxicity is negligible, and disinfection can only be achieved in the presence of the photocatalytic powder. For completeness, only a slight reduction (about 0.1 Log reduction) was observed for both dark controls concerning *S. aureus*. Consequently, the growth of this strain appears to be slightly reduced in the dark regardless of the presence of the photocatalytic nanomaterial. This effect is attributed to the strain’s growth behavior in the presence of light and is likely negligible.

Furthermore, the photocatalytic efficiency in the inactivation of the same starting concentration of bacteria (10^6^ CFU/mL) was monitored with both bacterial strains using a lower concentration of Fe-TiO_2_ nanoparticles, namely 0.5 g/L. Figure 3 shows that this lower dose of Fe-TiO_2_ nanoparticles resulted in decreased bacterial inactivation compared to experiments using 1 g/L. Indeed, only a 91% reduction, or 1.08 Log, was achieved (1.05 ± 0.05 and 1.12 ± 0.02 Log reductions for **E. coli** and *S. aureus*, respectively). Light control and dark control experiments were also carried out with the lower dose (0.5 g/L), and no significant Log reduction was observed (Figure S6). These results indicate that, under the employed experimental conditions, a higher dose of nanomaterial is necessary, as 1 g/L of Fe-TiO_2_ nanoparticles provides nearly three times more effective bacterial inactivation (3.4 and 3.1 for **E. coli** and *S. aureus*, respectively). Additionally, it is noteworthy that the decrease of viable bacteria at a lower concentration of Fe-TiO_2_ nanoparticles is comparable to that achieved by 1 g/L undoped TiO_2_.

**Figure 4:**
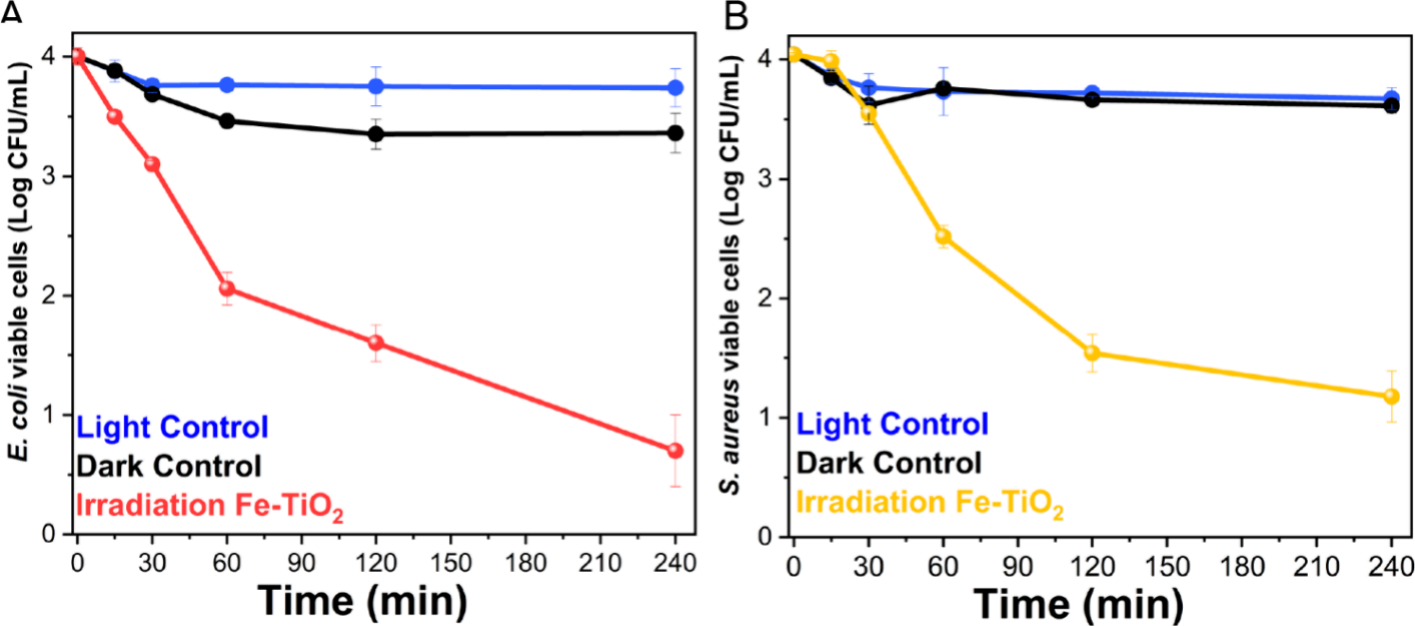
Evaluation of the viable CFU/mL over time (Log scale) for (A)**E. coli** and (B)*S. aureus* in the presence of 1 g/L Fe-TiO_2_ (yellow line) under visible light irradiation. An initial bacterial concentration of 10^4^ CFU/mL was used. All the panels also show the viable CFU/mL over time during the light control (blue line, bacteria suspension without photocatalyst under visible light irradiation) and dark control 1 (black line, bacterial suspension containing 1 g/L Fe-TiO_2_ in the dark).

Figure 4 presents the results of the disinfection experiments conducted with an initial bacteria concentration of 10^4^ CFU/mL for both **E. coli** and *S. aureus*. Given the findings from tests with higher bacterial concentrations, which indicated significantly lower activity of undoped TiO_2_, these photocatalytic tests were performed only with 1 g/L Fe-TiO_2_. The results showed a 99.9% (Log 3.31 ± 0.10) reduction for**E. coli** and 99.8% (Log 2.82 ± 0.11) for*S. aureus* following 240 min of light exposure. The purpose of these tests was to evaluate the effectiveness of the nanomaterial in the presence of a lower yet still substantial bacterial load.

Over time, the evaluation of the viable bacterial cells revealed trends like those reported in Figure 3, demonstrating an effective reduction in both strains. The controls indicated a slight decrease in the number of viable cells. Under these conditions, the mere presence of visible light (light control, blue curves) suggests that a minor physiological decrease in viable cells should be noted. This effect is observable in both strains (0.28 ± 0.15 and 0.42 ± 0.10 Log reduction for **E. coli** and *S. aureus*, respectively), but it is negligible compared to the impact of light in the presence of the photocatalyst. Following an initial decrease, the count remained nearly constant until the test concluded (up to 240 min). Simultaneously, some effects of powder toxicity (dark control 1, black curves) were recorded with both strains. **E. coli** showed a 0.7 ± 0.15 Log reduction of viable cells when kept in the dark with the nanomaterial powder present. *S. aureus*, on the other hand, exhibited a smaller decrease of viable cells in the dark (0.5 ± 0.09 Log reduction) compared to the Gram-negative **E. coli**. This trend further confirms our observations from the irradiated condition in this test (Figure 4) as well as in previous tests with a higher starting concentration of viable cells (10^6^ CFU/mL). At higher concentrations, viable cells were less affected by either the toxicity or photocatalytic properties of the nanometric powders, although these effects were still evident.

Then, the photocatalytic efficiency in inactivating the same initial concentration of bacteria (10^4^ CFU/mL) was tested with a lower concentration of Fe-TiO_2_ nanoparticles (0.5 g/L) using both bacterial strains. Once again, as shown in Figure S7, a lower dose of photocatalyst led to a reduced bacterial inactivation compared to the experiments carried out with 1 g/L. In fact, only a 92% reduction, or 1.22 Log, was reached (1.33 ± 0.13 and 1.12 ± 0.09 Log reductions for **E. coli** and *S. aureus*, respectively). However, the results indicate that at a higher dosage (1 g/L), the bacterial reduction is nearly three times greater as compared with a lower dosage (0.5 g/L). As illustrated in Figure S7, no such reduction was noted in the control tests at a lower dose (light control: blue curves, dark control 1: black curves). Furthermore, in this scenario, no toxic effects were recorded, as no difference in the viable bacterial amount is apparent in the dark control trend line.

It is important to note that the disinfection efficiency of Fe-TiO_2_ is slightly higher against the Gram-negative **E. coli** than against the Gram-positive *S. aureus*, as expected, as clearly shown with a 10^4^ CFU/mL starting concentration (Figure 4). A possible explanation is that this effect arises from the differing susceptibility of their cell membranes to the reactive oxygen species (ROS) produced during the photocatalytic process, as well as the unique characteristics of *S. aureus*. Indeed, it is known that ROS generated in aerated aqueous media during photocatalysis, mainly ^•^O_2_^–^ (superoxide anions) and ^•^OH (hydroxyl radicals) species, can penetrate and damage the cell membranes of bacteria, resulting in disinfection.^68,69^ Compared to **E. coli**, *S. aureus* has a peptidoglycan cell wall and an external polysaccharide capsule that better protects the microorganism.^37^ Additionally, it is catalase-positive;^36^ making it more likely to survive the ROS generated during the photocatalytic treatment.

Scheme 2 shows the events that may lead to the photocatalytic inactivation of **E. coli** and *S. aureus* after the photoactivation of Fe-TiO_2_ nanoparticles by visible light. Concerning the type of ROS present in our photocatalytic system, the photocatalytic behavior of the Fe-TiO_2_ nanoparticles has been studied in the presence of various radical scavengers under the same experimental conditions (specifically, a photocatalyst concentration of 1 g/L and illumination with visible light).^61^ It was found that superoxide species and positive holes (which trigger the formation of hydroxyl radicals in water) were the active species; thus, we expect that both ^•^O_2_^–^ and •OH are the primary ROS contributing to the disinfection activity of Fe-TiO_2_. Additionally, the presence of subsurface Fe^2+^ species (identified by XPS) strongly suggests that our synthesis procedure promotes the formation of Fe^2+^ species that, in turn, enhance the production of •O_2_^–^ ions, besides •OH radicals. Under illumination, Fe-TiO_2_ nanoparticles facilitate the formation of ROS naturally occurring in a nondeaerated aqueous phase. The visible light-activated Fe-TiO_2_ nanomaterial significantly reduced the number of viable cells, even in the presence of the defense strategies of the targeted bacteria.

Comparison with existing literature is challenging because experiments using different powders are conducted under various reaction conditions. Nevertheless, we chose to make some reasonable comparisons with a selection of studies presented in Table S1, which were performed under the most comparable conditions to ours, specifically two papers analyzing Fe-doped TiO_2_ powders with similar concentrations of photocatalyst (g/L) and bacteria (CFU/mL) under visible light.

For example, Yadav et al.^38^ examined various concentrations of photocatalytic materials (0.1, 0.5, 1, and 2 g/L) at a light intensity of 0.5 mW/cm^2^. They found that with 0.5 g/L of nanomaterial, only 60% of bacterial reduction was achieved after 240 min of treatment. In contrast, at 1 g/L, complete inactivation was reached, making it the most effective concentration. Indeed, increasing the photocatalytic dose beyond 1 g/L does not give any further advantage. Moreover, Khan et al.^34^ applied the same photocatalyst dose (1 g/L) to inactivate **E. coli** with an initial concentration of 10^4^ CFU/mL, attaining a 100% log reduction after 150 min. The faster disinfection they achieved is likely due to the higher intensity (500 W) of the visible light source used, compared to the intensity of our light source (7.3 W). On one hand, the results of bacterial inactivation reported here highlighted the significant efficiency of our Fe-TiO_2_ nanoparticles under visible light. On the other hand, the lack of comparable data underscores the need for further studies on this subject.

**Figure 5:**
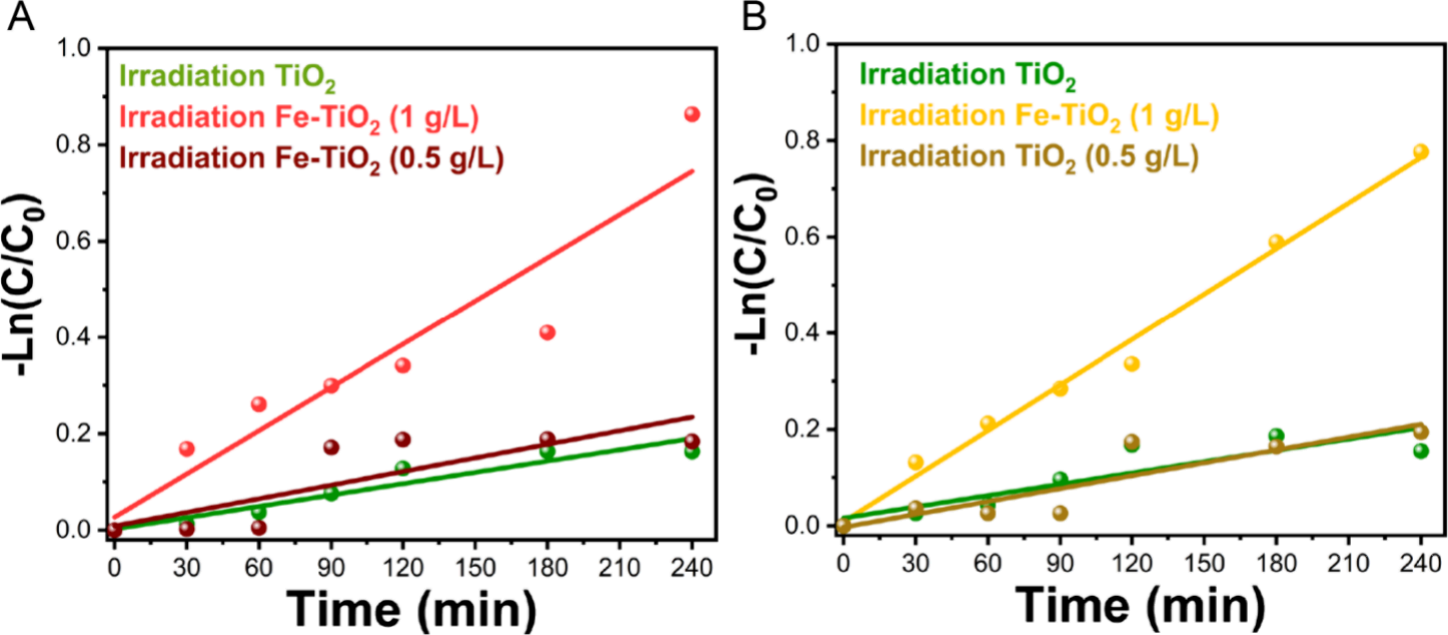
Kinetic curves of **E. coli** (A) and *S. aureus* (B) showing Log reduction under visible light irradiation in the presence of TiO_2_ (1 g/L) and Fe-TiO_2_ (1 and 0.5 g/L) with an initial bacterial concentration of 10^6^ CFU/mL.

The results obtained from these photocatalytic tests can be further investigated by examining the inactivation kinetics. Figure 5 reports the kinetics curves for disinfection with 10^6^ CFU/mL bacteria concentrations achieved with 0.5 g/L Fe-TiO_2_, 1 g/L Fe-TiO_2_, and undoped TiO_2_. With 1 g/L undoped TiO_2,_ the rates are nearly 3 times smaller than those obtained with 1 g/L Fe-TiO_2_. For the undoped material, a rate of approximately 0.8 × 10^–3^ ± 0.1 × 10^–3^ 1/min (*R*^2^ = 0.89) was calculated for **E. coli** and 0.8 × 10^–3^ ± 0.2 × 10^–3^ 1/min (*R*^2^ = 0.73) for *S. aureus*. With 1 g/L Fe-TiO_2_ rates of approximately 2.9 × 10^–3^ ± 0.5 × 10^–3^ 1/min (*R*^2^ = 0.87) and 3.1 × 10^–3^ ± 0.2 × 10^–3^ 1/min (*R*^2^ = 0.98) were calculated for **E. coli** and *S. aureus*, respectively. At the lower dosage of 0.5 g/L, Fe-TiO_2_, a rate of approximately 0.9 × 10^–3^ ± 0.2 × 10^–3^ 1/min (*R*^2^ = 0.61) was calculated for **E. coli** and ca. 0.8 × 10^–3^ ± 0.2 × 10^–3^ 1/min (*R*^2^ = 0.75) for *S. aureus*, indicating a rate very similar to that achieved with 1 g/L of undoped TiO_2_. For completeness, the kinetics for an initial bacterial concentration of 10^4^ CFU/mL are shown in Figure S8, which reveals slopes demonstrating an even faster rate (nearly twice as fast) compared to 10^6^ CFU/mL. Furthermore, comparing the slopes of the kinetic curves highlights the superior effectiveness of Fe-TiO_2_ in disinfecting Gram-negative **E. coli** compared to Gram-positive *S. aureus*.

To confirm that the bacteria were effectively killed by the photocatalytic treatment, live/dead fluorescence staining was performed. In this test, cells with an intact membrane stain green with SYTO 9, while cells with a compromised membrane that are dead or dying stain red with propidium iodide. Figures S9 and S10 show the corresponding microscope images. These images reveal that initially, live, green-stained cells were observed for both strains. After 15 min, in the presence of visible light-activated Fe-TiO_2_ nanoparticles, the number of red-stained cells increased, indicating bacterial inactivation. With prolonged exposure to the photocatalytic treatment, more red-stained bacterial cells of *S. aureus* and **E. coli** were observed in the case of Fe-TiO_2_ nanoparticles, likely due to cell membrane damage caused by the photogenerated ROS. Some green-stained cells of *S. aureus* could be seen after 240 min, confirming incomplete bacterial inactivation. However, with **E. coli**, almost all the cells were red-stained after 240 min, indicating that the photocatalytic treatment was more effective against **E. coli** than *S. aureus*.

### 3.3 Results of the Reusability Study

Following the initial inactivation experiments, a study was conducted to examine the stability and reusability of the Fe-TiO_2_ photocatalyst. Subsequently, after the initial tests of bacterial inactivation, the nanoparticles were recovered, as detailed in the experimental section, and reused for up to three cycles.

**Figure 6:**
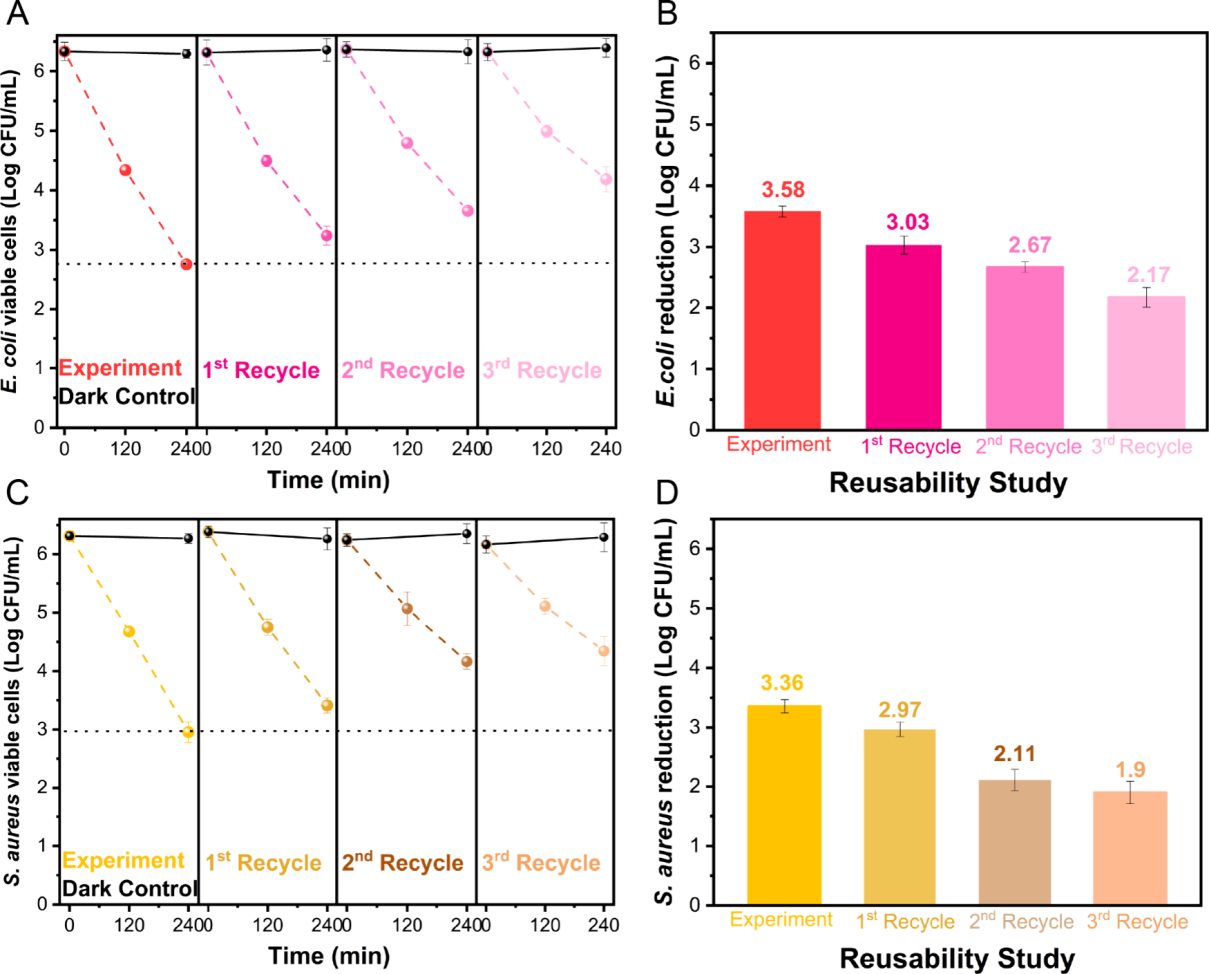
Reusability study of Fe-TiO_2_ nanoparticles for up to three cycles following the initial inactivation of both bacteria strains. Panels (A) and (B) report, respectively, the viable CFU/mL (Log scale) of **E. coli** and the total Log reduction of **E. coli** after 240 min in each cycle. Panels (C) and (D) report, respectively, the viable CFU/mL (Log scale) of *S. aureus* and the total Log reduction of *S. aureus* after 240 min in each cycle. All tests conducted under visible light irradiation were performed with an initial bacterial concentration of 10^6^ CFU/mL, in the presence of 1 g/L photocatalyst. The dark control 1 experiments (black lines) were carried out with a suspension containing 10^6^ CFU/mL bacteria and 1 g/L Fe-TiO_2_ in the dark.

The bacterial inactivation efficiency, expressed as Log reduction, is shown in Figure 6. During the first recycling cycle, the bacterial inactivation of both strains was comparable to what is reported in Figure 3, and bacterial inactivation continued to be observed in each subsequent cycle. A slight reduction was noted in the following recycling tests for both strains (Figure 6A,C). Nevertheless, the maximum Log reduction (illustrated in the bar chart of Figure 6B,D) decreases after each cycle, with an overall decrease of 0.86 Log and 1.07 Log after three recycling tests for **E. coli** and *S. aureus*, respectively; specifically, the efficiency against **E. coli** and *S. aureus* was (slightly) reduced by approximately 7 and 10%, respectively. The dark controls (bacterial suspension and Fe-TiO_2_ recycled nanoparticles in the dark) were also conducted in each recycling experiment, and no significant Log reduction was observed (Figure 6A,C, black lines).

Overall, the results of the reusability tests demonstrated that the Fe-TiO_2_ photocatalyst could be recovered and recycled after liquid applications, indicating that it is also a quite stable material. ICP-MS analysis carried out on the recovered powder (Table S3) measured an overall Fe content of 2.26 wt % after the reusability tests with **E. coli** and 2.18 wt % with *S. aureus*, which is lower than the original amount, likely due to some Fe leaching phenomena that could occur during the repeated washing steps. In comparison to the initial ICP-MS-determined Fe content of 2.44 wt %, however, a total Fe leaching of 7 and 11% was recorded after the reusability tests with **E. coli** and *S. aureus*, respectively, suggesting that optimizing the recycling conditions could yield even better results.

### 3.4 Photocatalytic Disinfection of an Actual Water Sample

**Figure 7:**
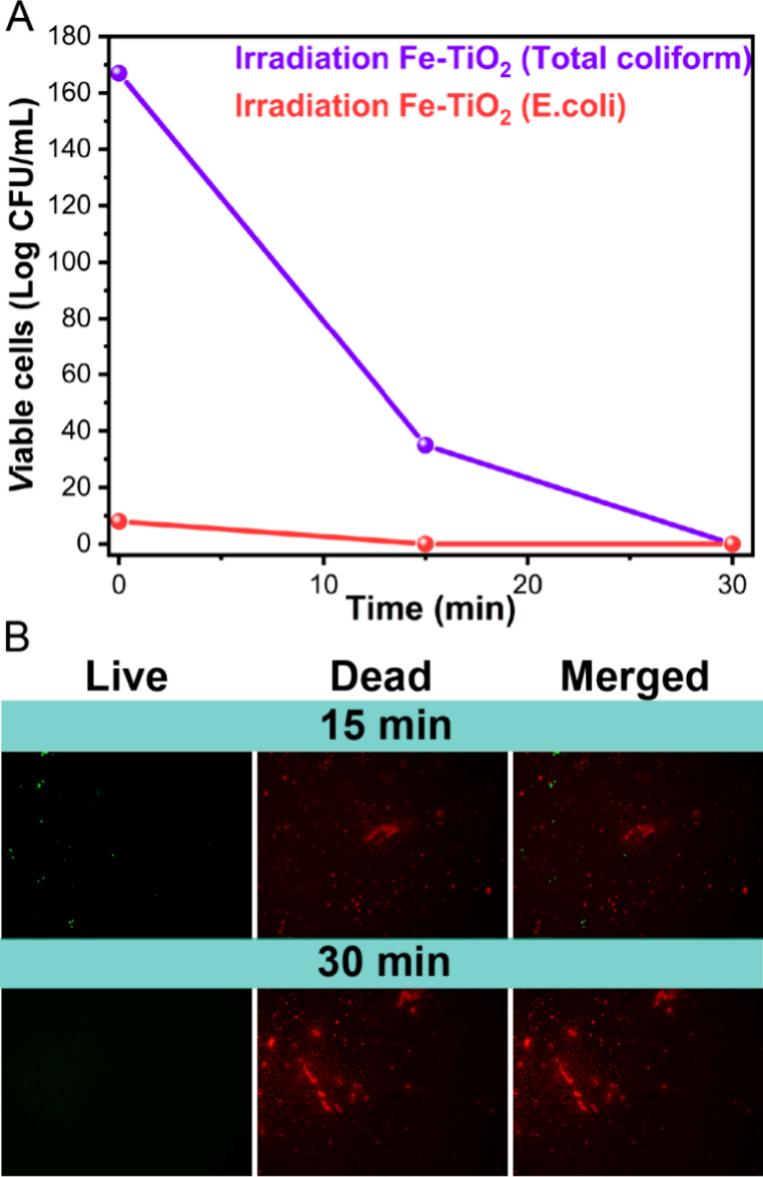
Photocatalytic disinfection of **E. coli** and *total coliforms* in an actual tap water sample (CFU/mL) using 1 g/L Fe-TiO_2_ nanoparticles activated by visible light (A). Fluorescence microscopy images of the live/dead staining that were taken during the photocatalytic disinfection test of the tap water sample over time (B).

An actual tap water sample was utilized to validate the bacterial disinfection properties of the Fe-TiO_2_ nanoparticles. The results of the analysis of the tap water sample are shown in Table S2, which includes the WHO’s threshold guidelines on drinking water quality for comparison.^6,70^ The physicochemical water quality parameters met WHO’s standards. However, the water quality parameters related to microbial contamination exceeded the threshold limits, with 167 CFU/mL detected for *total coliforms* and 8 CFU/mL for **E. coli** alone. This bacterial contamination in tap water likely resulted from some cross-contamination between the sewer lines and the drinking water network system. In any event, *total coliform, fecal coliform,* and **E. coli** are regarded as indicators of microbial contamination in drinking water. Therefore, the collected tap water was tested in the presence of 1 g/L of Fe-TiO_2_ nanoparticles to analyze the disinfection capability of this nanomaterial (Figure 7).

As shown in Figure 7A, 15 min of photocatalytic treatment led to a 0.9 Log (100%) reduction of **E. coli** and a 1.5 Log (77%) reduction of *total coliform*. After 30 min, a 100% reduction in *total coliforms* was observed. Selected fluorescence microscopy images related to the live/dead staining of samples taken during the test are presented in Figure 7B. After 15 min, a few green live cells coexist with some red dead cells, but after 30 min, no green fluorescence is visible, confirming extensive bacterial deactivation. Although the disinfection efficiency in tap water samples may be slightly higher than that achieved during disinfection experiments at both bacterial loads (10^6^ or 10^4^ CFU/mL), due to the lower viable bacteria present in the sample compared to the very high bacterial load tested earlier, the visual and qualitative observations from fluorescence microscopy affirm the excellent disinfection capability of the Fe-TiO_2_ nanomaterial, even with a real-life tap water sample.

## 4 Conclusions

Using a template-assisted sol–gel method, a Fe-doped TiO_2_ nanopowder with a nominal Fe content of 2.5 wt % was produced. This mesoporous nanomaterial exhibited a high specific surface area, the presence of 100% anatase phase, and consisted of roundish nanoparticles with relatively uniform shape and size.

In terms of optical properties in the visible range resulting from Fe-doping, the Fe-TiO_2_ nanoparticles were characterized by a bandgap of 2.80 eV, the presence of subsurface Fe^2+^ species, and some surface FeO_*x*_H_*y*_ species that absorb at 500 nm.

The photocatalytic activity under visible light was tested for antibacterial effectiveness against the Gram-negative **E. coli** and the Gram-positive *S. aureus* bacteria in liquid media, at relatively high bacteria concentrations of 10^6^ and 10^4^ CFU/mL, using two concentrations of photocatalyst: 1 and 0.5 g/L. Consistent with the literature, 1 g/L proved to be the optimal concentration for the experimental conditions used.

Compared to undoped TiO_2_ synthesized by the same method, the Fe-TiO_2_ nanoparticles proved to be more effective, likely due to their optical properties. They facilitated the photocatalytic disinfection of both **E. coli** and *S. aureus* under visible light illumination produced by an inexpensive, commercially available LED lamp. The bacterial Log reduction of **E. coli** (3.57 ± 0.21 Log) and *S. aureus* (3.49 ± 0.42 Log) achieved with 1 g/L of nanomaterial after 240 min under visible light irradiation in liquid media was verified through live/dead fluorescence staining. Reusability tests demonstrated that the Fe-TiO_2_ nanoparticles remained active for at least three cycles following the initial cycle, despite some limited Fe leaching.

The Fe-TiO_2_ nanoparticles were also tested under the same optimal conditions (1 g/L powder concentration and visible light illumination) to eliminate microbial contamination in an actual tap water sample collected from a household in Jamshoro, Pakistan. Interestingly, the bacteria were photocatalytically inactivated within 30 min of exposure under visible light irradiation.

Without comprehensive guidelines on the conditions to be used during such tests, comparing data obtained with different types of illumination, reactor configurations, and nanomaterials is not straightforward. However, a comparison with previous literature regarding microbial contamination due to the photocatalytic activity of Fe-doped TiO_2_ shows that the nanomaterial proposed here shows significant performance against high concentrations of both **E. coli** and *S. aureus* merely under visible light, which differs from most studies that focus on UV-light activation This suggests that the use of Fe-doped TiO_2_ nanoparticles for photocatalytic water disinfection under visible light warrants further studies. To implement a Fe-doped TiO_2_ photocatalyst in industrial or large-scale applications, producing 3D printed filters or membranes would benefit small and larger industries to inactivate pathogenic bacteria. These 3D filters and membranes can be utilized in the food industry, biomedical field, and water treatment. The advantages of these filters include sustainable recovery and recyclability options.

## Supporting Information Available

Summary of key literature studies on photocatalytic bacterial disinfection using Fe-doped TiO_2_ materials; water quality parameters of the contaminated tap water sample collected from a household in Jamshoro (Pakistan), and EDS and ICP-MS determined iron content in the Fe-TiO_2_ nanopowder before after four photocatalytic cycles with *E. coli* and *S. aureus*; additional experimental data concerning FESEM images of the two studied nanomaterials; HR XP spectrum for the Fe 2p line of the Fe-TiO_2_ nanopowder; selected HAADF maps and EDS spectra of the Fe-TiO_2_ nanopowder before after four photocatalytic cycles with *E. coli* and *S. aureus*; selected representative images of *E. coli* and *S. aureus* bacterial counts (CFU/mL) obtained with an initial bacterial concentration of 10^6^ CFU/mL under visible light irradiation in the presence of 1 g/L Fe-TiO_2_, undoped TiO_2_ and during control experiments; evaluation over time of the viable CFU/mL (Log scale) of *E. coli* and *S. aureus* obtained with an initial bacterial concentration of 10^6^ CFU/mL in the presence of 0.5 g/L Fe-TiO_2_ under visible light irradiation, during light control and dark control 1 experiments; evaluation over time of the viable CFU/mL (Log scale) of *E. coli* and *S. aureus* starting from an initial bacterial concentration of 10^4^ CFU/mL under visible light irradiation in the presence of 0.5 g/L Fe-TiO_2_, during light control and dark control 1 experiments; kinetic curves of *E. coli* and *S. aureus* under visible light irradiation in the presence of 0.5 and 1 g/L Fe-TiO_2_ with initial bacteria concentrations of 10^6^ CFU/mL and 10^4^ CFU/mL; and optical microscope images of live/dead fluorescence staining related to the photocatalytic disinfection of *S. aureus* and of *E. coli* under visible light using 1 g/L Fe-TiO_2_ with an initial bacteria concentration of 10^4^ CFU/mL (PDF)
