# Supplementary material for: Photocatalytic Disinfection of Selected Waterborne Pathogens by Visible Light-Active Nano Iron-Doped TiO2 Obtained by a Sol–Gel Method
Source: ACS Appl Nano Mater. 2025 Apr 9;8(19):10066–79. doi: 10.1021/acsanm.5c01408 (PMC12090347; doi:10.1021/acsanm.5c01408)
Supplement: Supplementary file 1 — an5c01408_si_001.pdf [file an5c01408_si_001.pdf]

Supporting Information

Photocatalytic Disinfection of Selected Waterborne Pathogens by Visible Light-active Nano Iron-Doped TiO_2 Obtained by a Sol-gel Method

*Najeebullah Channa[1], Tanveer A. Gadhi[2], Francesca Stefania Freyria[1,3], Alessandro Chiadò[1,4], Nicola Blangetti[1], Nicoletta Ditaranto,[5] Barbara Bonelli[1,3,4]**

[1] Department of Applied Science and Technology, Corso Duca degli Abruzzi 24, Politecnico di Torino,10129 Torino, Italy.

[2] US Pakistan Center for Advanced Studies in Water (USPCASW), Mehran University of Engineering and Technology, Jamshoro, 76062, Pakistan.

[3] INSTM-Unit of Torino Politecnico, Corso Duca degli Abruzzi 24, Politecnico di Torino,10129 Torino, Italy.

[4] Polito[BIO]Med Lab, Corso Duca degli Abruzzi 24, Politecnico di Torino,10129 Torino, Italy.

[5] Chemistry Department, Aldo Moro University of Bari, Via Orabona 4, Bari, 70126, Italy.

* Corresponding author: Barbara Bonelli, email: (barbara.bonelli@polito.it).

Table S1. Summary of key literature studies on photocatalytic bacterial disinfection using Fe-doped TiO_2 materials. The table includes details on the photocatalytic material (Photocatalyst), the method used to evaluate disinfection efficiency, the type of bacteria (EC stands for E. coli, SA stands for S. aureus) along with the strain, if reported, the initial bacterial concentration (C_{ibact}), the photocatalyst concentration (C_{ph}), the light source used (Type of Light Source), its range (Range), and the overall disinfection efficiency (Eff. %). N.A. indicates that the data is not available.

**Photocatalyst**	**Method**	**Bacteria**	**C_{ibact} (CFU/mL)**	**C_{ph} (g/L)**	**Type of Light Source**	**Range**	**Power (W) /lumens (ln)**	**Eff (%)**	**Ref.**
Fe-doped TiO_2	Plate count/ Live & Dead	EC (ATCC 8739) and SA (ATCC 25923)	10^6 - 10^4	0.5 and 1	White LED lamp (Philips E27)	Vis	7.3/1535	99.9	This Study
Fe-doped TiO_2	Plate count	EC & SA	10^8	1	Fluorescent lamps (Philips TLD 8)	Vis	(8×8=64)/(380 ×8=3040)	100	1
Fe-doped TiO_2	Plate count	EC (ATCC-25922)	10^4	1	Vis light source	Vis	500/10,000	100	2
Fe-doped TiO_2	Optical density	Resistant EC	N.A.	0.4	UV lamp	UV	(2 × 8)/(960×2)	95	3
Fe- doped TiO_2	Inhibition Zone	EC	N.A.	N.A.	Ambient visible light	N.A.	N.A.	N.A.	4
Fe-doped TiO_2/MWCNTs nanocomposite	Plate count	*Bacillus subtilis* & *Pseudom*	10^8	1	Fluorescent lamps (Philips TLD 8 W)	Vis	(8×8)/(380×8)	100	5

		onas aeruginosa							
Fe-doped TiO_2/activated carbon nanocomposite	Plate count	EC & SA	10^5	0.5	N.A.	Vis	N.A.	100	6
Cu/Fe/N -doped TiO_2	Zone of inhibition	EC (ATCC25922) and SA(ATCC29213) and	10^6	N.A.	N.A.	Vis	N.A.	N.A.	7
Fe/Al-doped TiO_2	Plate count	EC (CIP 54.127)	10^6	N.A.	XBO lamp	Vis	N.A.	99.9	8
Fe/Cd-doped TiO_2	Plate count	EC (ATCC 11105)	10^7	1	Tungsten-Halogen lamps	Vis	N.A.	100	9
Fe-doped TiO_2 hollow spheres	Plate count	EC (ATCC-25922)	10^6	0.2	Solar Simulator	Vis	300	70	10
Fe-doped TiO_2 thin film	Plate count	EC (ATCC 8739)	10^5	N.A.	Xenon lamp	Vis	500/15000	97.5	11
Fe-doped TiO_2 thin film	Plate count	EC	N.A.	N.A.	N.A.	Vis	N.A.	96 - 99	12
Fe-doped TiO_2 thin film	McFarland tube	EC & SA	10^8	N.A.	UV light	UV	8/N.A.	100 (EC) 97% (SA)	13
Fe-doped TiO_2/clay composite	Plate count	EC K-12	10^6	N.A.	N.A.	UV	NA	93	14

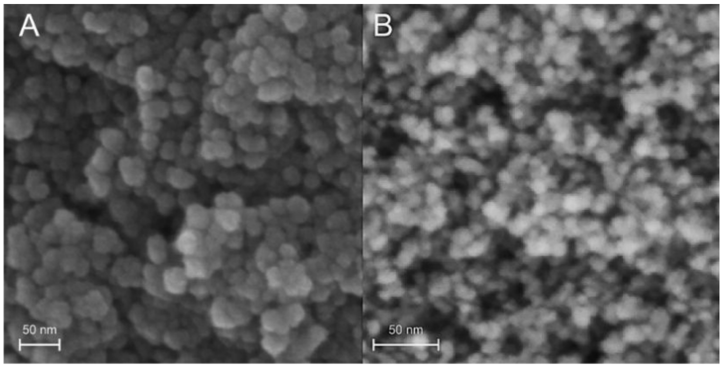

Figure S1. Selected FESEM images of undoped TiO_2 (A) and Fe-TiO_2 (B).

Table S2. Water quality parameters of the contaminated tap water sample collected from a household in Jamshoro, Pakistan.

Parameter	Unit	Measured value	WHO Limit
pH	-	7.9	6.5-8.5
Total Dissolved Solids (TDS)	mg/L	320	1000
Sulfate	mg/L	34	250
Nitrate	mg/L	2.3	50
Chloride	mg/L	75	250
Total hardness	mg/L	57	500
E. coli	CFU/mL	8	0
S. aureus	CFU/mL	0	100
Total coliforms	CFU/mL	167	0

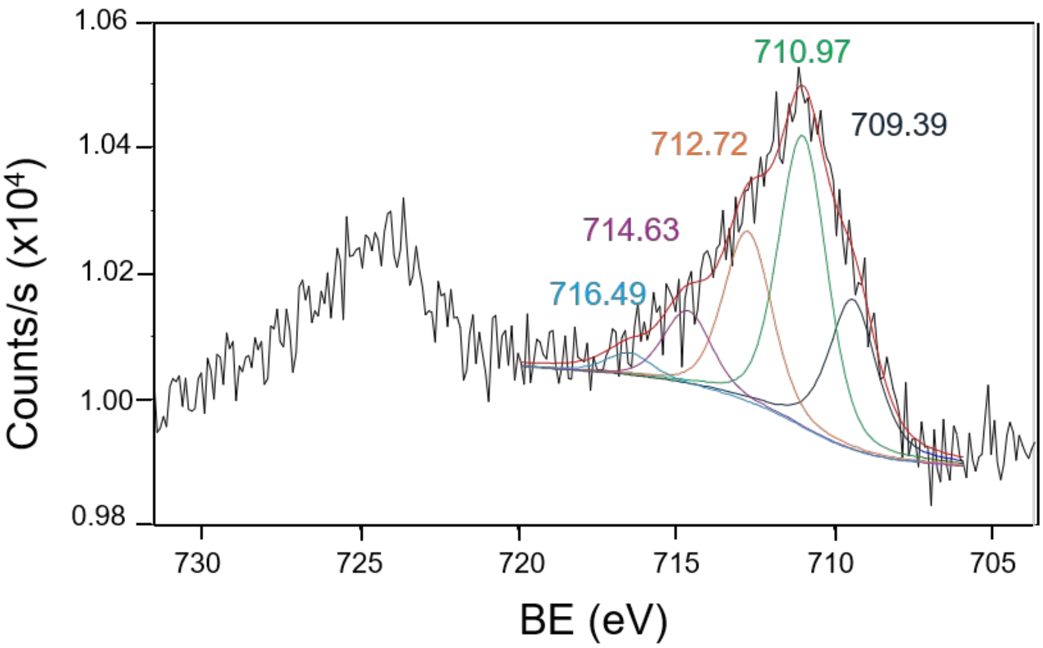

Figure S2. HR XP spectrum for the Fe2p line of the Fe-TiO_2 nanopowder.

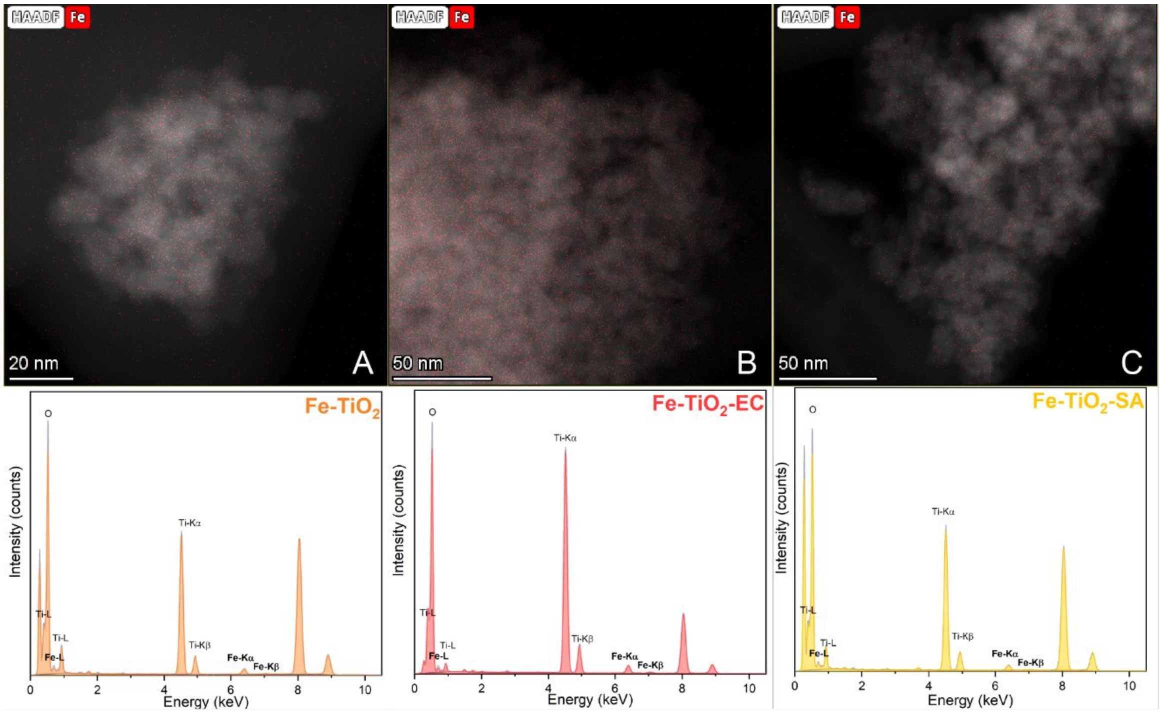

Figure S3. Selected HAADF map and EDS spectrum, labelling only the elements present in the nanopowder (element labels from the TEM grid or any potential adventitious elements have been

omitted). Panel A: Fe-TiO_2 nanopowder before use. Panel B: Fe-TiO_2 nanopowder after four photocatalytic cycles with *E. coli* (EC). Panel C: Fe-TiO_2 nanopowder after four cycles with *S. aureus* (SA).

Table S3. EDS[a] and ICP-MS[b] determined iron content in the Fe-TiO_2 nanopowder, before and after four photocatalytic cycles involving *E. coli* (EC) and *S. aureus* (SA).

**Sample**	**Fe content (wt. %)[a]**	**Fe content (wt. %)[b]**
Fe-TiO_2	2.56 ±0.41	2.44
Fe-TiO_2-EC	2.50 ±0.49	2.26
Fe-TiO_2-SA	2.49±0.39	2.18

[a]Iron content as determined from at least two HAADF map area images.

In **Table S3**, the slight discrepancy between the two values may be due to EDS being a semi-quantitative technique, while ICP-MS is a more reliable method for element quantification.

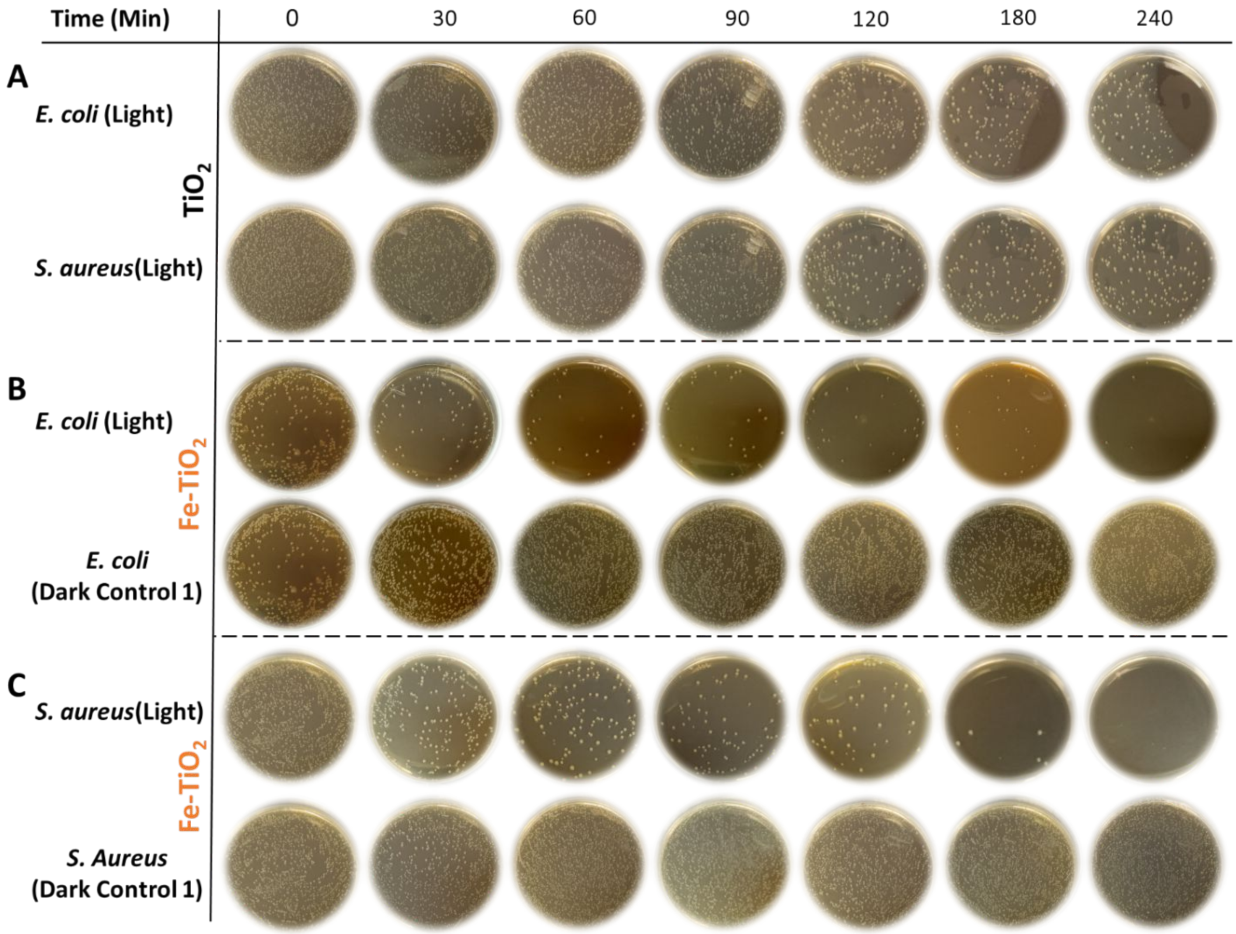

Figure S4. Selected representative images of *E. coli* and *S. aureus* bacterial counts (CFU/mL) at various dilutions of the sample (ranging from 10^{-1} to 10^{-4}) and different treatment times, were obtained with an initial bacterial concentration of 10^6 CFU/mL in the presence of 1 g/L Fe-TiO_2. Panel A: photocatalytic disinfection tests (*E. coli* and *S. aureus*) conducted with undoped TiO_2 under visible light irradiation. Panel B: photocatalytic disinfection tests of *E. coli* with Fe-TiO_2 under visible light irradiation and dark control 1. Panel C: photocatalytic disinfection tests of *S. aureus* with Fe-TiO_2 under visible light irradiation and dark control 1.

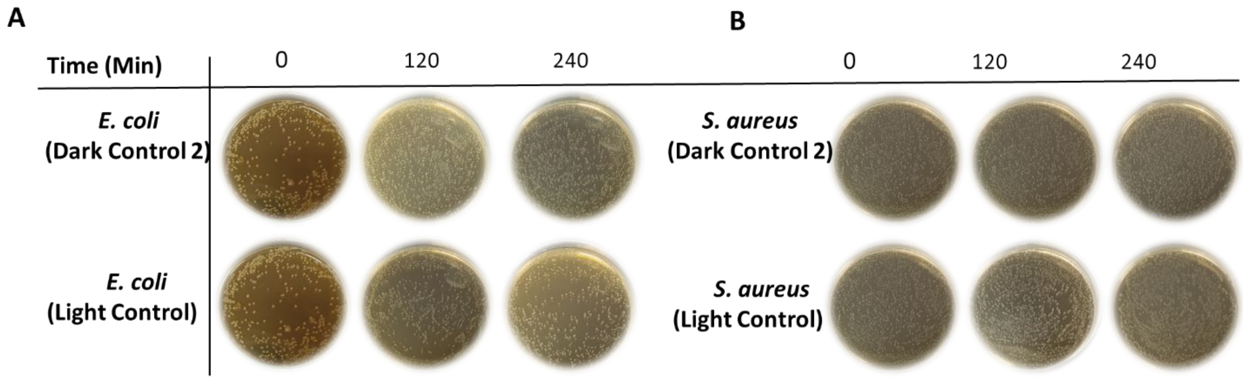

Figure S5. Selected representative images of the plates used for CFU/mL evaluation over time, obtained at a 100 dilution of the sample during light control and dark control 2 experiments, conducted with a starting bacterial concentration of 10^6 CFU/mL for *E. coli* (panel A) and *S. aureus* (panel B). Light control experiments involve a bacterial suspension without any photocatalyst under visible light irradiation; dark control 2 experiments involve a bacterial suspension without any photocatalyst in the dark.

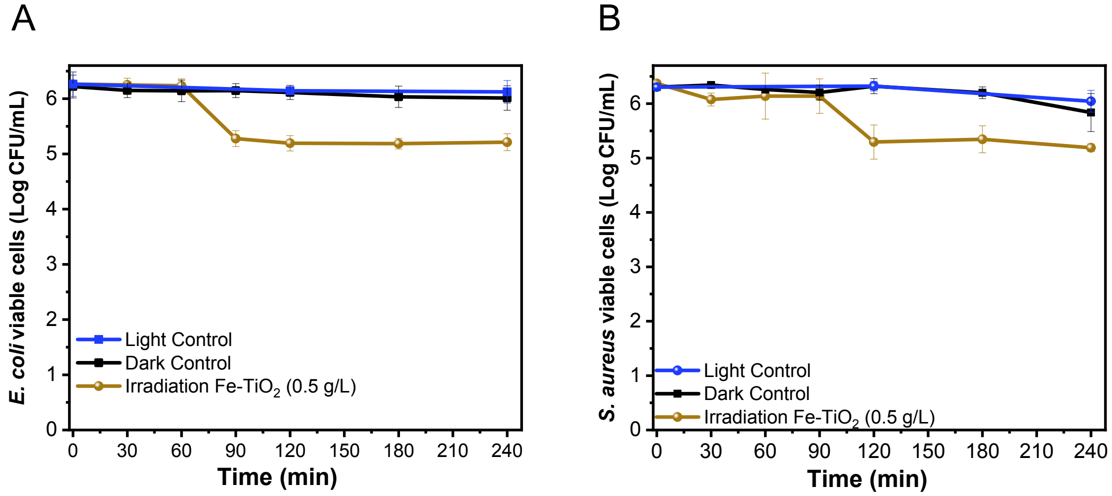

Figure S6. Evaluation over time of the viable CFU/mL (Log scale) of *E. coli* (panel A) and *S. aureus* (panel B) with 0.5 g/L Fe-TiO_2 under visible light irradiation (brown line), starting from an initial bacterial concentration of 10^6 CFU/mL. The panels also report the viable CFU/mL over time

of the light control experiments (i.e., bacterial suspension without the photocatalyst under visible light irradiation, blue line), and the dark control 1 experiments (bacterial suspension containing 0.5 g/L Fe-TiO_2 in the dark, black line).

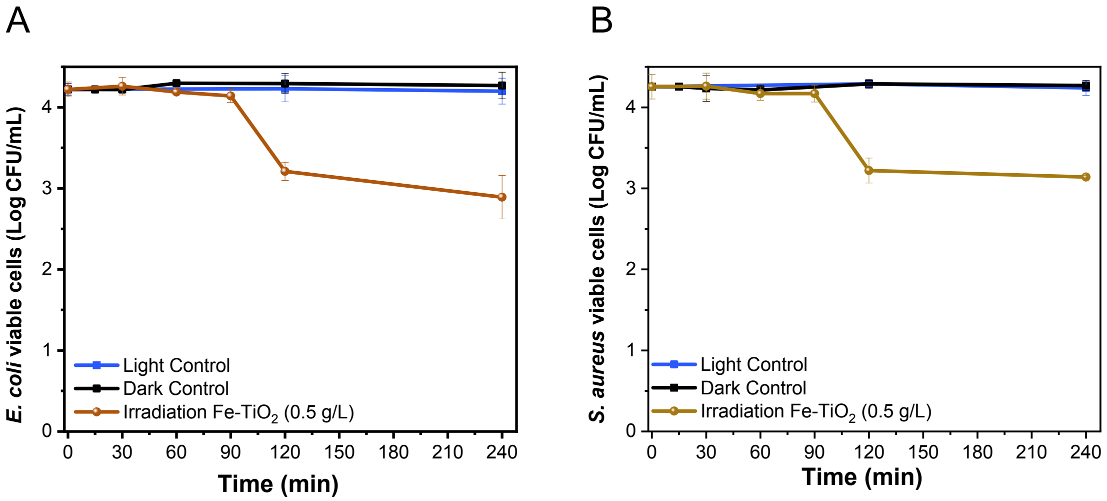

Figure S7. Evaluation over time of the viable CFU/mL (Log scale) of *E. coli* (panel A) and *S. aureus* (panel B) with 0.5 g/L Fe-TiO_2 under visible light irradiation (brown line), starting from an initial bacterial concentration of 10^4 CFU/mL. The panels also report the viable CFU/mL over time of the light control experiments (i.e., bacteria suspension without the photocatalyst under visible light irradiation, blue line), and of the dark control 1 experiments (bacteria suspension in the presence of 0.5 g/L Fe-TiO_2 in the dark, black line).

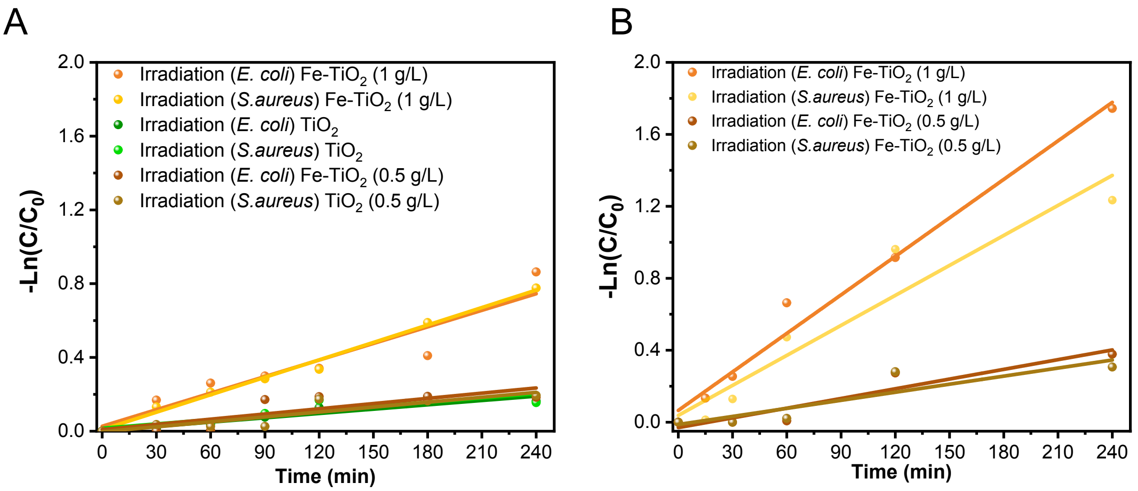

Figure S8. Kinetic curves of *E. coli* and *S. aureus* showing Log reduction under visible light irradiation in the presence of 0.5 and 1 g/L Fe-TiO_2 with an initial bacteria concentration of 10^6 CFU/mL (panel A) and 10^4 CFU/mL (panel B).

Figure S8 compares the kinetic curves obtained in the presence of Fe-TiO_2 at concentrations of 0.5 and 1 g/L with 10^6 CFU/mL (panel A) and 10^4 CFU/mL (panel B) as starting concentrations of bacteria. When starting with a lower concentration of viable microorganisms (panel B), the rate of disinfection was even faster than that obtained with 10^6 CFU/mL (panel A), measuring approximately $7.1\times10^{-3} \pm 0.5 \times10^{-3}$ 1/min (R^2=0.98) for *E. coli* and $5.5\times10^{-3} \pm 0.8 \times10^{-3}$ 1/min (R^2=0.92) for *S. aureus*, when 1 g/L of nanopowder was used. A similar trend was observed with a lower dose of 0.5 g/L at a lower initial concentration of 10^4 CFU/mL, yielding a rate of about $1.8 \times10^{-3} \pm 0.3 \times10^{-3}$ 1/min (R^2=0.85) for *E. coli* and approximately $1.5 \times10^{-3} \pm 0.4 \times10^{-3}$ 1/min (R^2=0.75) for *S. aureus*. The kinetics at this lower dose exhibited almost the same rate as the

undoped nanoparticles. This confirms the enhancement of the photocatalytic performance of the nanomaterial under visible light.

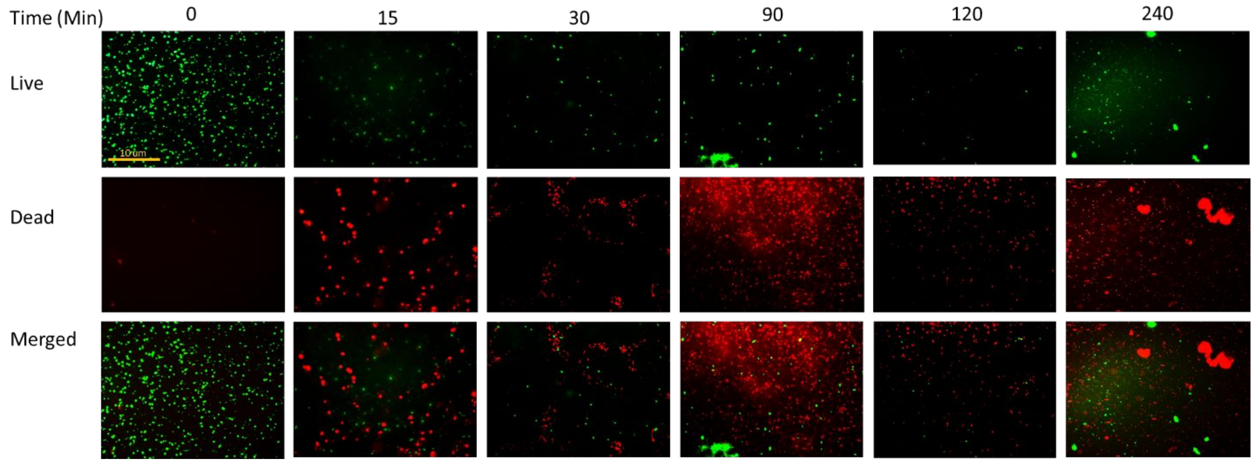

Figure S9. Optical microscope images of live/dead fluorescence staining (green: live bacterial cells; red: dead bacterial cells) related to the photocatalytic disinfection of *S. aureus* under visible light using 1 g/L Fe-TiO_2 with an initial bacteria concentration of 10^4 CFU/mL, after 0, 15, 30, 90, 120 and 240 minutes.

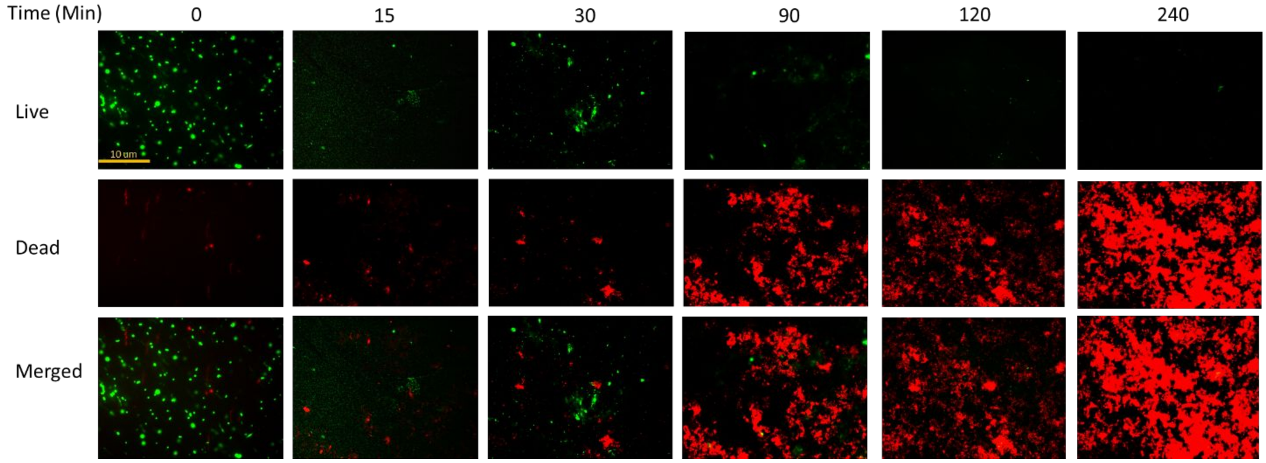

Figure S10. Optical microscope images of live/dead fluorescence staining (green: live bacterial cells; red: dead bacterial cells) related to the photocatalytic disinfection of *E. coli* under visible light using 1 g/L Fe-TiO_2 with an initial bacteria concentration of 10^4 CFU/mL, after 0, 15, 30, 90, 120 and 240 minutes.

Bacterial inactivation was monitored using a live/dead viability kit for fluorescence microscopy. The corresponding images are presented in **Figures S9** and **S10** for *S. aureus* and *E. coli*, respectively. At the start (0 min), predominantly green (live) bacterial cells were observed, with very few red-stained cells. During the photocatalytic treatment, the viability of the green cells decreased while the number of red cells increased accordingly.

References

(1) Yadav, H. M.; Kolekar, T. V.; Pawar, S. H.; Kim, J. S. Enhanced Photocatalytic Inactivation of Bacteria on Fe-Containing TiO_2 Nanoparticles under Fluorescent Light. *J Mater Sci Mater Med* **2016**, *27* (3), 1–9. https://doi.org/10.1007/S10856-016-5675-8.

(2) Khan, M. S.; Shah, J. A.; Riaz, N.; Butt, T. A.; Khan, A. J.; Khalifa, W.; Gasmi, H. H.; Latifee, E. R.; Arshad, M.; Al-Naghi, A. A. A.; Ul-Hamid, A.; Arshad, M.; Bilal, M. Synthesis and Characterization of Fe-TiO_2 Nanomaterial: Performance Evaluation for RB5 Decolorization and In Vitro Antibacterial Studies. *Nanomaterials 2021, Vol. 11, Page 436* **2021**, *11* (2), 436. https://doi.org/10.3390/NANO11020436.

(3) Poostforooshan, J.; Belbekhouche, S.; Olszok, V.; Stodt, M. F. B.; Simmler, M.; Bierwirth, M.; Nirschl, H.; Kiefer, J.; Fritsching, U.; Weber, A. P. Synthesis of Pure and Fe-Doped TiO_2

Nanoparticles via Electrospray-Assisted Flame Spray Pyrolysis for Antimicrobial Applications. *ACS Appl Nano Mater* **2023**. https://doi.org/10.1021/ACSANM.3C03107.

(4) Kamble, R. J.; Gaikwad, P. V. Peroxy Titanium Complex Derived Fe-Doped TiO_2 Nanoparticles: Synthesis, Properties and Antibacterial Activity. *Mater Today Proc* **2021**, *45*, 3784–3788. https://doi.org/10.1016/J.MATPR.2021.01.282.

(5) Koli, V. B.; Delekar, S. D.; Pawar, S. H. Photoinactivation of Bacteria by Using Fe-Doped TiO_2-MWCNTs Nanocomposites. *J Mater Sci Mater Med* **2016**, *27* (12), 1–10. https://doi.org/10.1007/S10856-016-5788-0.

(6) Baruah, M.; Ezung, S. L.; Supong, A.; Bhomick, P. C.; Kumar, S.; Sinha, D. Synthesis, Characterization of Novel Fe-Doped TiO_2 Activated Carbon Nanocomposite towards Photocatalytic Degradation of Congo Red, E. Coli, and S. Aureus. *Korean Journal of Chemical Engineering* **2021**, *38* (6), 1277–1290. https://doi.org/10.1007/S11814-021-0830-4.

(7) Yin, J.; Lv, L.; Chu, Y.; Tan, L. Highly Antibacterial Cu/Fe/N Co-Doped TiO_2 Nanopowder under Visible Light. *Inorg Chem Commun* **2023**, *151*, 110587. https://doi.org/10.1016/J.INOCHE.2023.110587.

(8) Schlur, L.; Begin-Colin, S.; Gilliot, P.; Gallart, M.; Carré, G.; Zafeiratos, S.; Keller, N.; Keller, V.; André, P.; Greneche, J. M.; Hezard, B.; Desmonts, M. H.; Pourroy, G. Effect of Ball-Milling and Fe-/Al-Doping on the Structural Aspect and Visible Light Photocatalytic Activity of

TiO_2 towards Escherichia Coli Bacteria Abatement. *Materials Science and Engineering: C* **2014**, *38* (1), 11–19. https://doi.org/10.1016/J.MSEC.2014.01.026.

(9) Feilizadeh, M.; Mul, G.; Vossoughi, M. E. Coli Inactivation by Visible Light Irradiation Using a Fe–Cd/TiO_2 Photocatalyst: Statistical Analysis and Optimization of Operating Parameters. *Appl Catal B* **2015**, *168–169*, 441–447. https://doi.org/10.1016/J.APCATB.2014.12.034.

(10) Ang, J. K. K.; Chua, J. S. M.; Chang, Z. J.; Li, Z.; Bai, H.; Sun, D. D. An Ion Exchange Approach Assembled Multi-Dimensional Hierarchical Fe–TiO_2 Composite Micro-/Nano Multi-Shell Hollow Spheres for Bacteria Lysis through Utilizing Visible Light. *Catal Sci Technol* **2018**, *8* (8), 2077–2086. https://doi.org/10.1039/C8CY00078F.

(11) Meng, D.; Liu, X.; Xie, Y.; Du, Y.; Yang, Y.; Xiao, C. Antibacterial Activity of Visible Light-Activated TiO_2 Thin Films with Low Level of Fe Doping. *Advances in Materials Science and Engineering* **2019**, *2019*. https://doi.org/10.1155/2019/5819805.

(12) Arellano, U.; Asomoza, M.; Ramírez, F. Antimicrobial Activity of Fe–TiO_2 Thin Film Photocatalysts. *J Photochem Photobiol A Chem* **2011**, *222* (1), 159–165. https://doi.org/10.1016/J.JPHOTOCHEM.2011.05.016.

(13) AL-Jawad, S. M. H.; Taha, A. A.; Salim, M. M. Synthesis and Characterization of Pure and Fe Doped TiO_2 Thin Films for Antimicrobial Activity. *Optik (Stuttg)* **2017**, *142*, 42–53. https://doi.org/10.1016/J.IJLEO.2017.05.048.

(14) Thakur, I.; Verma, A.; Rmeci, B. O. Fe−TiO_2 Composite Mediated the Hybrid Effect of Photocatalysis and Photo-Fenton for the Inactivation of Escherichia Coli Using a Continuous Flow Recirculation Reactor. *Ind. Eng. Chem. Res* **2021**, *60*, 7558–7571. https://doi.org/10.1021/acs.iecr.1c00628.
